# Global, regional and national burden of chronic obstructive pulmonary disease over a 30‐year period: Estimates from the 1990 to 2019 Global Burden of Disease Study

**DOI:** 10.1111/resp.14349

**Published:** 2022-08-23

**Authors:** Hao‐Yang Li, Teng‐Yu Gao, Wei Fang, Chen‐Yang Xian‐Yu, Nian‐Jia Deng, Chao Zhang, Yu‐Ming Niu

**Affiliations:** ^1^ Center for Evidence‐Based Medicine and Clinical Research, Taihe Hospital Hubei University of Medicine Shiyan China; ^2^ Department of Stomatology & Center for Evidence‐Based Medicine and Clinical Research Gongli Hospital of Shanghai Pudong New Area Shanghai China

**Keywords:** chronic obstructive pulmonary disease, disability adjusted life‐years, global burden of disease, incidence, mortality

## Abstract

**Background and objective:**

Chronic obstructive pulmonary disease (COPD) is the most prevalent chronic respiratory disease. This study investigated the global, regional and country burden of COPD based on gender, age and socio‐demographic indices (SDIs) in the last 30‐year period from 1990 to 2019.

**Methods:**

The COPD data, including incidence, mortality and disability‐adjusted life years (DALYs), were obtained from the 2019 Global Burden of Disease Study. If age‐standardized incidence rate (ASIR) or death rate (ASDR) remains almost constant or decreases, the number of cases will still increase as the global population increases substantially. Estimated annual percentage change (EAPC) was calculated to assess incidence, mortality and DALY trends.

**Results:**

The incidence of COPD increased by 85.89% from 8,722,966 cases in 1990 to 16,214,828 cases in 2019, and the ASIR decreased from 216.48/100,000 persons in 1990 (95%UI, 204.56–227.33) to 200.49 per 100,000 persons (95%UI, 188.63–212.57) in 2019. The ASIR increased (EAPC = 0.05, 95%CI, 0.01–0.10) in the low SDI region, was stable in the high SDI region, and fell in the other three SDI regions. Men had a higher ASIR than women over the past 30 years, and there were differences in the incidence rates for different age groups. Male mortality and DALYs were higher than female mortality. ASDR decreased by 2.13% (95%CI, −2.23% to −2.02%) per year and the annual age‐standardized DALY rate decreased by 1.97% (95%CI, −2.05% to −1.89%).

**Conclusions:**

The ASIR, ASDR and age‐standardized DALY rate of COPD declined overall in the last 30 years, and were highest in the low‐middle SDI region.

## Graphical abstract

Over the last 30 years, the absolute number of COPD‐associated deaths and DALYs increased significantly worldwide, but the ASIR, ASDR and age‐standardized DALY rate of COPD declined. Health authorities and policymakers must take these differences into account to make better use of limited resources.

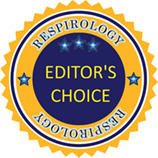

**See related** Editorial

## INTRODUCTION

The World Health Organization (WHO) stated on 22 June 2021 that chronic obstructive pulmonary disease (COPD) is the third leading cause of death globally, causing 3.23 million deaths in 2019.^1^ COPD is a leading cause of chronic morbidity and mortality worldwide. Although a variety of interventions have been applied to the prevention and treatment of COPD, it remains one of the major contributors to the Global Burden of Disease (GBD).^2^, ^3^

COPD is a preventable and treatable disease characterized by incomplete reversible airflow obstruction. As the population ages, COPD will become more prevalent.^4^, ^5^ In recent years, most studies on the burden of COPD disease have been regional or national,^6^, ^7^ and some studies have examined the global burden of COPD prevalence.^4^, ^8^, ^9^ These studies suggest that the burden of COPD will increase in the coming years, and risk factors affect different populations differently. However, there were few studies on the global burden of COPD incidence, mortality, disability‐adjusted life years (DALYs), estimated annual percentage change (EAPC) and factors of death/DALY risk in countries and regions around the world. This study is more comprehensive, representative and referential.

The DALY value can be estimated by adding years of disabled life (YLDs) and years of life lost (YLLs).^10^ If the EAPC value and its lower limit of 95% confidence interval (CI) were both positive, it was considered a significant increasing trend for the age‐standardized incidence/death/DALY rate (ASR). If the EAPC value and its upper limit of 95% CI were both negative, it was considered a significant decreasing trend for the ASR. Otherwise, ASR was considered to be stable. If the EAPC was statistically significant but the uncertainty intervals of the GBD estimates overlap, ASR was still considered stable.^11^

This study aimed to investigate the incidence, mortality, DALYs and the corresponding trends of COPD in the last 30 years according to sex, age, socio‐demographic index (SDI) and country. Furthermore, the relativity of risk factors on COPD mortality and DALYs were investigated.

## METHODS

### Data resources

Information on the annual incidence rate, death rate, DALY and risk factors of COPD was obtained from the GBD 2019 study (http://ghdx.healthdata.org/gbd-results-tool). The GBD 2019 study includes data on 369 diseases and injuries in 204 countries and territories,^12^ and data on age and gender were also obtained to assess their impact on COPD. The location information was classified according to three criteria to investigate the global burden of COPD.

The SDI is used as a generalized measure to reflect the development of each region in relation to health outcomes in the GBD study. It is the geometric average of the total fertility rate of the population under 25 years old, the average education level of the population over 15 years old and the distribution of per capita income. Based on the SDI values in 2019, 204 countries were divided into five quintiles: high, high‐middle, middle, low‐middle and low,^3^, ^13^ and specific SDI for 204 countries is available in the GBD 2019 Study (https://ghdx.healthdata.org/record/ihme‐data/gbd‐2019‐socio‐demographic‐index‐sdi‐1950‐2019). Differences in the COPD incidence rate, death rate, DALY rate and corresponding trends in 204 countries and territories were calculated from 1990 to 2019.

### Statistical analysis

Trends in COPD incidence and mortality were assessed using age‐standardized incidence rate (ASIR), age‐standardized death rate (ASDR), age‐standardized DALY and the corresponding EAPCs. ASIR/ASDR can be obtained by comparing the age structure of different age groups or the age structure of the same population over time. The purpose of using ASIR/ASDR is to exclude the age component from affecting the incidence or mortality of the population, as a result of which the incidence or mortality of disease varies greatly at different ages.

ASR (per 100,000 population) is equal to the sum of the product of the specific age ratio ($a_{i}$) in age group i and the number (or weight) ($w_{i}$) of the selected reference standard population group i divided by the sum of number (or weight) of the standard population, that is, $\mathrm{ASR}=\frac{\sum_{i=1}^{A}a_{i}w_{i}}{\sum_{i=1}^{A}w_{i}}\times 100,000.$ ^11^, ^14^ EAPC was applied to reflect trends in ASR (age‐standardized incidence/death/DALY rate) over specific time intervals: *y* = *α* + *βx* + *ε*, where *y* is ln (ASR), *x* represented the calendar year. EAPC = 100 × (exp(*β*) − 1), and its 95%CI was also obtained from this model. If the ASIR or ASDR of a disease is almost constant or decreasing, the number of cases will still increase as the global population increases year by year. This is because the global population has increased far more than the rate has changed. In addition, to observe the relationship between EAPC and ASR/SDI, the Pearson's correlation coefficient^15^ and corresponding scatter plots were employed. The 2019 Human Development Index can be used as a proxy for the level of health care in each country. All data analyses and calculations were performed using R Software (version 4.1.0).

## RESULTS

### Change in incidence of COPD

#### At the global level

**FIGURE 1:**
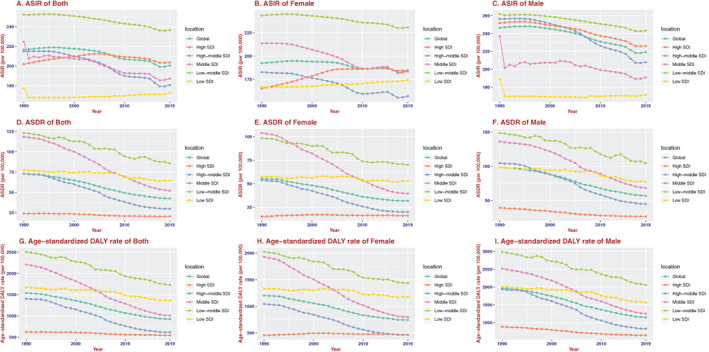
The change trends of age‐standardized incidence, death and DALY rate among different SDI quintiles. (A) Age‐standardized both incidence. (B) Age‐standardized female incidence. (C) Age‐standardized male incidence. (D) Age‐standardized both death rate. (E) Age‐standardized female death rate. (F) Age‐standardized male death rate. (G) Age‐standardized both DALY rate. (H) Age‐standardized female DALY rate. (I) Age‐standardized male DALY rate

**FIGURE 2:**
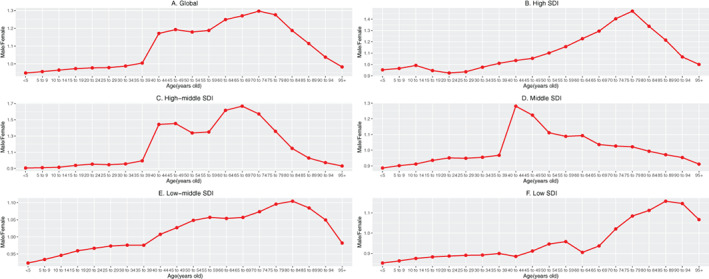
The ratio of male to female incidence among different age groups in 2019. (A) Global. (B) High SDI. (C) High‐middle SDI. (D) Middle SDI. (E) Middle‐low SDI. (F) Low SDI. SDI, socio‐demographic index.

There were 16,214,828 (95% uncertainty interval [UI]; 15,224,111–17,220,809) incidences of COPD in 2019 and 8,722,966 (95% UI; 8,242,714–9,166,892) incidences in 1990. Additionally, the annual incidence increased significantly and almost doubled (Table 1 and Figure S1A in the Supporting Information). Contrary to the 85.89% increase in incident cases over the past 30 years, the ASIR decreased from 216.48/100,000 persons (95% UI, 204.56–227.33) in 1990 to 200.49/100,000 persons (95% UI, 188.63–212.57) in 2019 (Table 1 and Figure S1B in the Supporting Information). Men had a higher ASIR than women over the past 30 years (Figure 1A). There were differences in the incidence rates of men and women in different age groups. The male/female ratio of the 35–39 year age group reached 1.00, and the ratio of the 40–44 year age group rapidly increased to nearly 1.17, and reached a peak in the 70–74 year age group (Figure 2).

**TABLE 1:** The incident cases and ASIR in 1990 and 2019 and its temporal trends

	1990		2019		1990–2019
	Incident cases no. * 10^2^(95%UI)	ASIR per 100,000 no.(95%UI)	Incident cases no. * 10^2^(95%UI)	ASIR per 100,000 no.(95%UI)	EAPC no.(95%CI)
Overall	87.23(82.43–91.67)	216.48(204.56–227.33)	162.15(152.24–172.21)	200.49(188.63–212.57)	−0.33(−0.38 to −0.29)
Sex					
Female	41.54(39.26–43.22)	192.30(181.52–202.42)	79.32(74.36–84.31)	184.00(172.72–195.34)	−0.22(−0.26 to −0.17)
Male	45.69(43.22–47.98)	246.36(233.03–257.87)	82.83(77.78–88.05)	219.30(206.58–232.04)	−0.48(−0.53 to −0.42)
Socio‐demographic index					
High SDI	20.39(19.22–21.42)	201.82(191.26–211.66)	36.09(34.32–37.71)	203.69(194.53–212.17)	0.02(−0.05–0.08)
High‐middle SDI	22.46(21.12–23.71)	215.01(202.90–226.02)	35.36(32.87–38.04)	180.61(168.81–193.32)	−0.71(−0.77 to −0.64)
Middle SDI	23.73(22.26–25.12)	224.68(210.29–237.24)	44.89(41.47–48.57)	187.27(173.62–201.43)	−0.51(−0.57 to −0.45)
Low‐middle SDI	15.88(15.16–16.51)	252.36(240.47–261.70)	32.42(30.66–34.02)	236.58(223.91–247.68)	−0.26(−0.28 to −0.23)
Low SDI	4.73(4.50–4.97)	177.33(168.14–186.00)	10.02(9.50–10.56)	172.62(163.03–181.52)	0.05(0.01–0.1)

Abbreviations: ASIR, age‐standardized incidence rate; CI, confidence interval; EAPC, estimated annual percentage change; SDI, sociodemographic indices; UI, uncertainty interval.

#### SDI level analysis

**FIGURE 3:**
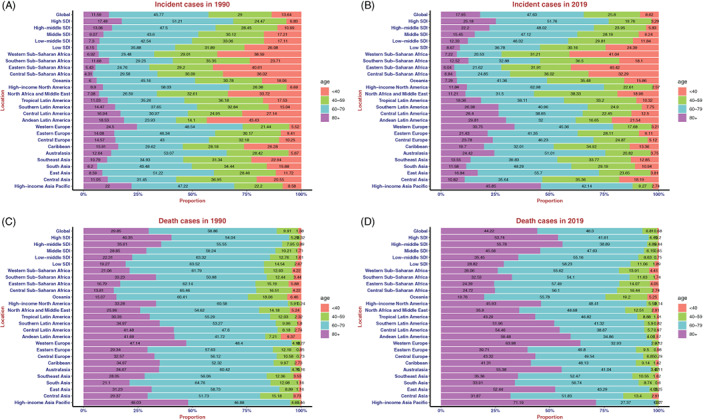
Distribution of different ages in chronic obstructive pulmonary disease incidence/death cases by region. (A) Incidence in 1990. (B) Incidence in 2019. (C) Death rate in 1990. (D) Death rate in 2019

As shown in Table 1 and Figure S2A in the Supporting Information, ASIR increased in the low SDI region, was stable in the high SDI region, and decreased in the other three SDI regions. The ASIR of women increased in the high SDI and low SDI regions and decreased in the other three SDI regions. The ASIR of men was stable in the low SDI region and decreased in the other four SDI regions. In addition, there was a negative correlation between EAPC and the ASIR (*ρ* = −0.55, *p* < 0.01, Figure S3A in the Supporting Information) and a non‐significant correlation between EAPC and SDI (Figure S3B in the Supporting Information). Figure 3A,B shows that a lower SDI was consistently associated with higher rates of young incident cases and lower rates of older incident cases. The proportion of annual incident cases under 60 years old decreased year by year, whereas the proportion of annual incident cases over 60 years old increased year by year (Figure S4A in the Supporting Information).

#### At the GBD regional and national level

The ASIR showed an upward trend in most regions and countries (Table S1 and Figures S5–S9 in the Supporting Information).

### Change in deaths due to COPD

#### At the global level

There were 3,280,636 (95% UI; 2,902,855–3,572,367) deaths in 2019 and 2,520,219 (95% UI; 2,118,059–2,719,388) deaths in 1990 (Table 2). The number of deaths in 2019 was 30.17% higher than that in 1990, whereas the ASDR significantly decreased, with an EAPC of −2.13 (95% CI, −2.23 to −2.02). It dropped from 7298/100,000 people (95% UI; 6155–7875) in 1990 to 4252/100,000 people (95% UI, 4631–3763) in 2019 (Table 2 and Figure S1C in the Supporting Information). Men had slightly higher ASDR than women over the past 30 years (Figure 1B). The major factor of female and male global COPD risk of death was smoking (Figures S10A, S11A in the Supporting Information).

**TABLE 2:** The death cases and ASDR in 1990 and 2019 and its temporal trends

	1990		2019		1990–2019
	Death cases no. * 10^2^(95% UI)	ASDR per 100,000 no.(95% UI)	Death cases no. * 10^2^(95% UI)	ASDR per 100,000 no.(95% UI)	EAPC no.(95% CI)
Overall	25.20(21.18–27.19)	72.98(61.55–78.75)	32.81(29.03–35.72)	42.52(46.31–37.63)	−2.13(−2.23 to −2.02)
Sex					
Female	10.95(82.17–12.32)	55.32(41.67–62.13)	13.99(11.40–16.02)	31.84(36.46–25.95)	−2.21(−2.31 to −2.10)
Male	14.25(12.50–15.67)	98.44(85.97–107.67)	18.81(16.96–20.71)	56.80(51.17–62.51)	−2.13(−2.24 to −2.02)
Socio‐demographic index					
High SDI	2.57(2.40–2.82)	24.01(22.33–26.43)	4.40(3.85–4.82)	20.38(18.01–22.23)	−0.71(−0.78 to −0.65)
High‐middle SDI	6.68(5.31–7.27)	72.99(57.77–79.67)	5.84(5.15–7.03)	29.76(26.19–35.75)	−3.58(−3.77 to −3.39)
Middle SDI	9.00(7.08–9.92)	118.54(94.28–129.80)	10.40(9.17–11.63)	52.27(46.05–58.70)	−3.13(−3.28 to −2.97)
Low‐middle SDI	5.57(4.76–6.26)	123.11(106.10–138.55)	9.64(8.02–10.89)	86.07(71.71–97.03)	−1.32(−1.40 to −1.24)
Low SDI	1.37(1.09–1.59)	77.15(60.90–90.35)	2.52(2.14–2.87)	64.43(54.44–73.33)	−0.67(−0.77 to −0.56)

Abbreviations: ASIR, age‐standardized incidence rate; CI, confidence interval; EAPC, estimated annual percentage change; SDI, sociodemographic indices; UI, uncertainty interval.

#### SDI level analysis

As shown in Table 2 and Figure S2B in the Supporting Information, ASDR decreased in all five SDI regions. The ASDR of women was stable in the high SDI region and decreased in the other four SDI regions. In addition, there was a negative correlation between EAPC and ASDR (*ρ* = −0.29, *p* < 0.01, Figure S3C in the Supporting Information) and a non‐significant correlation between EAPC and SDI (Figure S3D in the Supporting Information). Interestingly, we also found that in 1990, the lower the SDI, the lower the proportion of deaths in people older than 80 years, and the higher the proportion in people aged less than 80 years. By 2019, this pattern reversed, with a higher proportion of deaths among people over 80 years of age in the high‐middle SDI region than in the high SDI region (Figure 3C,D).

The proportion of annual deaths under 80 years old decreased year by year, whereas the proportion of annual incidence cases over 80 years old increased year‐by‐year (Figure S4B in the Supporting Information). It is worth mentioning that the mortality rate of 95+ female was higher than the male mortality rate in the middle SDI region (Figure S12 in the Supporting Information). The major factor for risk of COPD death among women was ambient particulate matter in the middle SDI region (Figure S10D in the Supporting Information) and household air pollution in the low‐middle SDI and low SDI regions (Figure S10E,F in the Supporting Information).

#### At the GBD regional and national level

The ASDR showed a declining trend in most regions and countries (Table S1 and Figures S5–S9 in the Supporting Information).

### Change in DALYs of COPD

#### At the global level

There were 74,432,367 (95% UI; 68,204,127–80,193,347) COPD‐associated DALYs in 2019 and 59,241,939 (95% UI; 51,208,419–63,591,584) DALYs in 1990 (Table 3). The number of DALYs in 2019 was 25.64% higher than that in 1990, whereas DALYs significantly decreased, with an EAPC of −1.97 (95%CI, −2.05 to −1.89), dropping from 153,772/100,000 people (95%UI; 133,069–164,746) in 1990 to 92,608/100,000 people (95%UI; 84,876–99,767) in 2019 (Table 3 and Figure S1D in the Supporting Information). Men had slightly higher DALYs than women over the past 30 years (Figure 1C). The major factor of female and male global COPD DALYs risk was smoking (Figures S13A and S14A in the Supporting Information).

**TABLE 3:** The DALY and age‐standardized DALY rate in 1990 and 2019 and its temporal trends

	1990		2019		1990–2019
	DALY no. * 10^3^(95% UI)	Age‐standardized DALY rate per 100,000 no.(95% UI)	DALY no. * 10^3^(95% UI)	Age‐standardized DALY rate per 100,000 no.(95% UI)	EAPC no.(95% CI)
Overall	592.42(512.08–635.92)	1537.72(1330.69–1647.46)	744.32(682.04–801.93)	926.08(848.76–997.67)	−1.97(−2.05 to −1.89)
Sex					
Female	254.51(201.04–285.82)	1202.45(957.98–1348.05)	323.76(283.70–357.93)	744.12(652.18–822.20)	−1.90(−1.98 to −1.82)
Male	337.91(294.78–370.81)	1972.42(2154.33–1736.84)	420.56(383.71–461.50)	1149.12(1050.15–1257.26)	−2.07(−2.15 to −1.98)
Socio‐demographic index					
High SDI	65.30(60.83–69.44)	622.94(579.97–662.16)	103.26(94.55–110.87)	543.53(499.18–582.88)	−0.57(−0.61 to −0.52)
High‐middle SDI	142.98(119.01–154.94)	1405.33(1161.51–1521.66)	122.96(112.10–141.50)	617.69(562.78–708.90)	−3.29(−3.45 to −3.12)
Middle SDI	206.10(165.97–226.19)	2207.64(1778.71–2412.00)	229.13(208.86–252.30)	1007.20(915.70–1112.45)	−2.98(−3.09 to −2.87)
Low‐middle SDI	139.54(121.50–154.79)	2504.97(2177.41–2776.72)	221.49(190.30–246.18)	1728.64(1488.81–1923.71)	−1.35(−1.41 to −1.29)
Low SDI	38.34(32.82–43.67)	1666.94(1378.03–1894.34)	67.18(59.37–74.72)	1364.11(1201.64–1522.56)	−0.72(−0.81 to −0.63)

Abbreviations: ASIR, age‐standardized incidence rate; CI, confidence interval; EAPC, estimated annual percentage change; SDI, sociodemographic indices; UI, uncertainty interval.

#### SDI level analysis

Regarding SDI level analysis, as shown in Figure S2C in the Supporting Information and Table 3, DALYs decreased in all five SDI regions. The DALYs of women were stable in the high SDI region and decreased in the other four SDI regions. In addition, there was a negative correlation between EAPC and DALY (*ρ* = −0.33, *p* < 0.01, Figure S3E in the Supporting Information) and a non‐significant correlation between EAPC and SDI (Figure S3F in the Supporting Information). As shown in Figure S15E in the Supporting Information, the DALY rate of both men and women peaked for the 85–89 year age group in 1990 and then began to decline, whereas the DALY rate of women increased with age in 2019. In the high SDI region, high‐middle SDI and middle SDI regions, the ratio of the male to female age‐standardized DALY rate tended to decline after the 85–89 year age group (Figure 16 in the Supporting Information). In the middle SDI region, the major factor of female DALY risk was ambient particulate matter (Figure S13D in the Supporting Information). In the low‐middle SDI and low SDI regions, the major factor of female DALY risk was household air pollution, followed by ambient particulate matter (Figure S13E,F in the Supporting Information).

#### At the GBD regional and national level

The age‐standardized DALY rate showed a declining trend in most regions and countries (Table S1 and Figures S5–S9 in the Supporting Information).

## DISCUSSION

Over the 30‐year period from 1990 to 2019, the ASIR, ASDR and age‐standardized DALY rate of COPD worldwide decreased by 7.39%, 41.74% and 39.78%, respectively. This phenomenon mainly reflects positive measures, including environmental governance, novel therapeutic measures^16^, ^17^ and reduction of the misdiagnosis rate.^18^, ^19^

Overall, the incidence of COPD was higher in North America, Oceania, Asia and most of Europe, except Eastern Europe. However, the incidence of COPD was lower in Eastern Europe and Africa.^20^ Considering environmental pollution, Asia and Africa had relatively serious PM 2.5 pollution.^21^ However, only Asia had a high incidence of COPD, but Africa had a low incidence. COPD was often difficult to diagnose^22^ in Africa because of poor medical care and lack of diagnostic equipment.

In the low SDI regions, the incidence of COPD in older adults gradually increased. The reason may be that the higher the SDI level was, the higher the level of medical care in this region, and people paid more attention to the maintenance of their health. However, the important finding was that the COPD incidence among the elderly in the low SDI region was the lowest of the five SDI regions, which may be attributed to the low diagnostic rates in the low SDI region. The proportion of incidences and deaths in young adults decreased year by year, whereas the proportion of incidences and deaths in older adults increased.

The mortality of COPD was higher in Asia and Papua New Guinea.^23^ The trends of DALYs and death were similar in COPD patients because the weight of YLD caused by COPD was not high, mainly YLLs.^24^, ^25^ Interestingly, the ratio of male to female DALYs in the low SDI region did not decline among older adults, as in the other four SDI regions. In 2019, the DALYs of females globally decreased compared with that in 1990, but the age distribution of DALYs was more concentrated in the elderly population.

Smoking was the main risk factor for COPD in males of all regions, but other risk factors, such as household and ambient air pollution, had a significant impact on females across Asia and Africa. Smoking was the most important reason for the high incidence of COPD in developed countries, such as North America.^8^, ^26^ Smoking in higher SDI regions caused a high proportion of deaths and DALYs, and there was no household air pollution among the risk factors in high SDI region. However, the proportion of deaths and DALYs caused by household air pollution and ambient particulate matter in the low SDI regions was relatively larger. In much of Asia and Oceania, indoor and outdoor ambient air pollution caused COPD disability rates much higher than the high‐income regions and developed countries.

This study inevitably had some limitations. First, GBD studies only consider regions and countries as basic units, without studying the influence of racial and ethnic factors on COPD. Second, SDI levels had also changed in different countries over 30 years. This was attributed to the country's overall education level and social security measures. These multifaceted factors were uncontrollable. Third, there were no detailed global PM 2.5 data over the years, which can only be supported by other literature.

In conclusion, the number of COPD cases, deaths and DALYs increased significantly worldwide in the last 30‐year period, but the age‐standardized incidence, mortality and DALY rates of COPD declined. The ASIR, ASDR and DALY rates were highest in the low‐middle SDI region, in both males and females. Hence, the low‐middle SDI region needs to pay more attention to the prevention and treatment of COPD and invest more in research and healthcare to reduce the incidence and mortality of COPD, and to implemented earlier effective intervention where possible. Countries in the middle SDI, low‐middle SDI and low SDI regions should improve diagnostic screening for COPD to identify patients earlier and prolong their lives.

## AUTHOR CONTRIBUTION

**Hao‐Yang Li:** Data curation (equal); formal analysis (equal); investigation (equal); methodology (equal); resources (equal); software (equal); validation (equal); writing – original draft (equal). **Teng‐Yu Gao:** Data curation (equal); formal analysis (equal); methodology (equal); resources (equal); software (equal); validation (equal). **Wei Fang:** Data curation (equal); formal analysis (equal); methodology (equal); project administration (equal); software (equal); supervision (equal). **Chen‐Yang Xian‐Yu:** Data curation (equal); formal analysis (equal). **Nian‐Jia Deng:** Data curation (equal); formal analysis (equal). **Chao Zhang:** Conceptualization (lead); investigation (equal); project administration (equal); resources (equal); software (equal); supervision (equal); visualization (equal); writing – review and editing (equal). **Yu‐Ming Niu:** Conceptualization (equal); data curation (equal); investigation (equal); methodology (equal); project administration (equal); visualization (equal); writing – original draft (equal).

## CONFLICT OF INTEREST

None declared.

## HUMAN ETHICS APPROVAL DECLARATION

Not applicable.

## Supporting information

Supporting Information

## Data Availability

The datasets generated and/or analysed in the current study are available from the Global Health Data Exchange query tool (http://ghdx.healthdata.org/gbd-results-tool).
