# Supplementary material for: Global, regional and national burden of chronic obstructive pulmonary disease over a 30‐year period: Estimates from the 1990 to 2019 Global Burden of Disease Study
Source: Respirology. 2022 Aug 23;28(1):29–36. doi: 10.1111/resp.14349 (PMC10087739; doi:10.1111/resp.14349)

Supplementary Materials

Supplementary Table S1 Three countries with the largest and lowest number of incidence, death, or DALY.

Supplementary Figure S1 The incident cases (A), age-standardized incidence (B), death (C) and DALY (D) rates of chronic obstructive pulmonary disease between 1990 and 2019 among sexes.

Supplementary Figure S2 The EAPC of chronic obstructive pulmonary disease ASR from 1990 to 2019, by sex and region.

Note: A: The EAPC of ASIR; B: The EAPC of ASDR; C: The EAPC of age-standardized DALY rate.

Supplementary Figure S3 The correlation between EAPC and Chronic Obstructive Pulmonary Disease ASR (incidence (a), death (c), and DALY (e)) in 1990 and HDI (incidence (b), death (d), and DALY (f)) in 2019.

Note: The circles represent countries that were available on SDI data. The size of circle represents the number of Chronic Obstructive Pulmonary Disease patients. The ρ indices Pearson's correlation coefficient and p values were derived from Pearson's correlation analysis. ASR, age-standardized incidence/death/DALY rate; EAPC, estimated annual percentage change; SDI, socio-demographic index

Supplementary Figure S4 The proportion of different ages in chronic obstructive pulmonary disease incidence (A) and death (B) by years.

Supplementary Figure S5 The global EAPC of chronic obstructive pulmonary disease for both sexes in 204 countries.

Note: A: The EAPC of ASIR; B: The EAPC of ASDR; C: The EAPC of age-standardized DALY rate.

Supplementary Figure S6 The global EAPC of chronic obstructive pulmonary disease for Female in 204 countries.

Note: A: The EAPC of ASIR; B: The EAPC of ASDR; C: The EAPC of age-standardized DALY rate.

Supplementary Figure S7 The global EAPC of chronic obstructive pulmonary disease for male in 204 countries.

Note: A: The EAPC of ASIR; B: The EAPC of ASDR; C: The EAPC of age-standardized DALY rate.

Supplementary Figure S8 The global disease burden of chronic obstructive pulmonary disease for Female in 204 countries.

Note: A: The ASIR of Chronic Obstructive Pulmonary Disease in 2019; B: The ASDR of chronic obstructive pulmonary disease in 2019; C: The age-standardized DALY rate of chronic obstructive pulmonary disease in 2019. ASIR: age-standardized incidence rate; ASDR: age-standardized death rate.

Supplementary Figure S9 The global disease burden of chronic obstructive pulmonary disease for male in 204 countries.

Note: A: The ASIR of chronic obstructive pulmonary disease in 2019; B: The ASDR of chronic obstructive pulmonary disease in 2019; C: The age-standardized DALY rate of chronic obstructive pulmonary disease in 2019. ASIR: age-standardized incidence rate; ASDR: age-standardized death rate.

Supplementary Figure S10 Causes of deaths risk for Female in global (A), high SDI (B), high-middle SDI (C), middle SDI (D), middle-low SDI (E) and low SDI (F).

Note: SDI, socio-demographic index.

Supplementary Figure S11 Causes of deaths risk for male in global (A), high SDI (B), high-middle SDI (C), middle SDI (D), middle-low SDI (E) and low SDI (F).

Note: SDI, socio-demographic index.

Supplementary Figure S12 The ratio of male to female ASDR among different age groups in global (A), high SDI (B), high-middle SDI (C), middle SDI (D), middle-low SDI (E) and low SDI (F).

Note: SDI, socio-demographic index. ASDR, age-standardized death rate.

Supplementary Figure S13 Causes of DALYs risk for Female in global (A), high SDI (B), high-middle SDI (C), middle SDI (D), middle-low SDI (E) and low SDI (F).

Note: SDI, socio-demographic index.

Supplementary Figure S14 Causes of DALYs risk for male in global (A), high SDI (B), high-middle SDI (C), middle SDI (D), middle-low SDI (E) and low SDI (F).

Note: SDI, socio-demographic index.

Supplementary Figure S15 The incidence, death, and DALY rates of Chronic Obstructive Pulmonary Disease in different age groups.

Note: A: Incidence in 1990. B: Incidence in 2019. C: Death rate in 1990. D: Death rate in 2019. E: DALY rate in 1990. F: DALY rate in 2019.

Supplementary Figure S16 The ratio of male to female age-standardized DALY rate among different age groups in global (A), high SDI (B), high-middle SDI (C), middle SDI (D), middle-low SDI (E) and low SDI (F).

Note: SDI, socio-demographic index.

Supplementary Table S1 Three countries with the largest and lowest number of incidence, death, or DALY.

Measure	Sex	Top three countries			Bottom three countries		
**2019ASR(per 100,000 people)**							
**ASIR**							
	Both	Papua New Guinea(341.01)	Democratic People's Republic of Korea(317.05)	Myanmar(306.74)	Kuwait(54.48)	Iraq(58.80)	Antigua and Barbuda(61.95)
	Female	Papua New Guinea(327.20)	Democratic People's Republic of Korea(319.93)	Denmark(305.15)	Trinidad and Tobago(40.61)	Saint Vincent and the Grenadines(40.99)	Kuwait(41.46)
	Male	Papua New Guinea(355.62)	Myanmar(326.80)	Democratic People's Republic of Korea(319.00)	Burkina Faso(89.46)	Turkmenistan(89.93)	Grenada(90.92)
**ASDR**							
	Both	Nepal(182.48)	Papua New Guinea(145.04)	Democratic People's Republic of Korea(105.20)	Japan(7.41)	Barbados(8.27)	Kuwait(8.35)
	Female	Nepal(168.43)	Papua New Guinea(131.67)	Bhutan(105.98)	Japan(3.98)	Bermuda(4.38)	Montenegro(4.48)
	Male	Nepal(198.53)	Papua New Guinea(159.70)	Democratic People's Republic of Korea(149.90)	Peru(10.38)	Kuwait(10.84)	Antigua and Barbuda(11.78)
**Age Standardized DALY Rate**							
	Both	Nepal(3318.41)	Papua New Guinea(2902.74)	Solomon Islands(2178.62)	Barbados(177.68)	Antigua and Barbuda(178.16)	Peru(189.63)
	Female	Nepal(3126.90)	Papua New	Bhutan(2008.19)	Bermuda(113.47)	Japan(122.44)	Trinidad and

			Guinea(2787.55)				Tobago(124.93)
	Male	Nepal(3528.42)	Papua New Guinea(3019.75)	Solomon Islands(2724.70)	Peru(209.68)	Antigua and Barbuda(222.80)	Barbados(232.94)

Supplementary Figure S1 The incident cases (A), age-standardized incidence (B), death (C) and DALY (D) rates of chronic obstructive pulmonary disease between 1990 and 2019 among sexes.

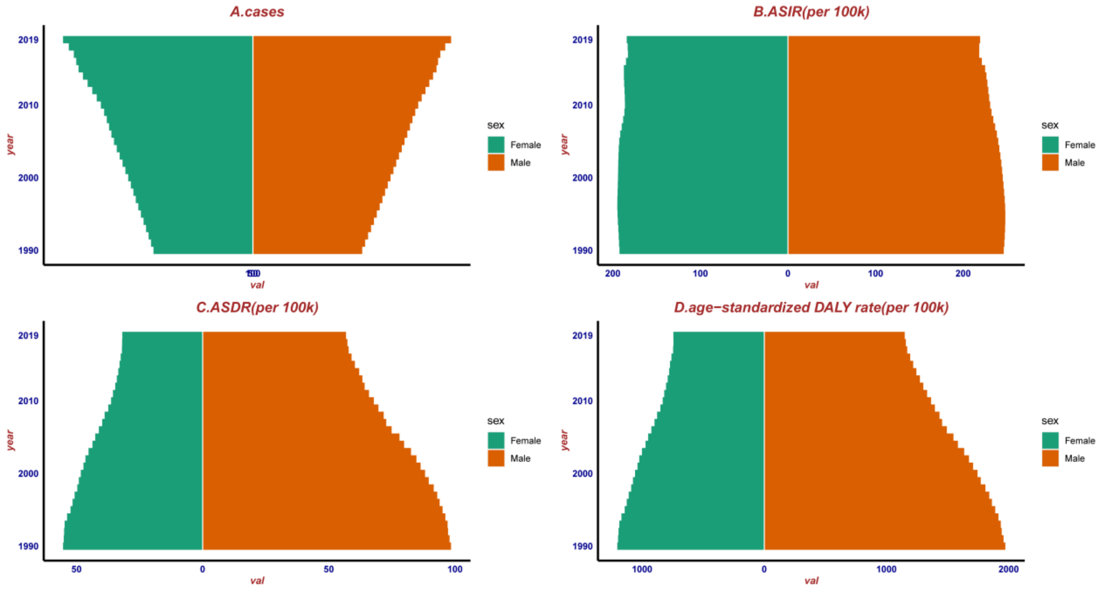

Supplementary Figure S2 The EAPC of Chronic Obstructive Pulmonary Disease ASR from 1990 to 2019, by sex and region.

Note: A: The EAPC of ASIR; B: The EAPC of ASDR; C: The EAPC of age-standardized DALY rate.

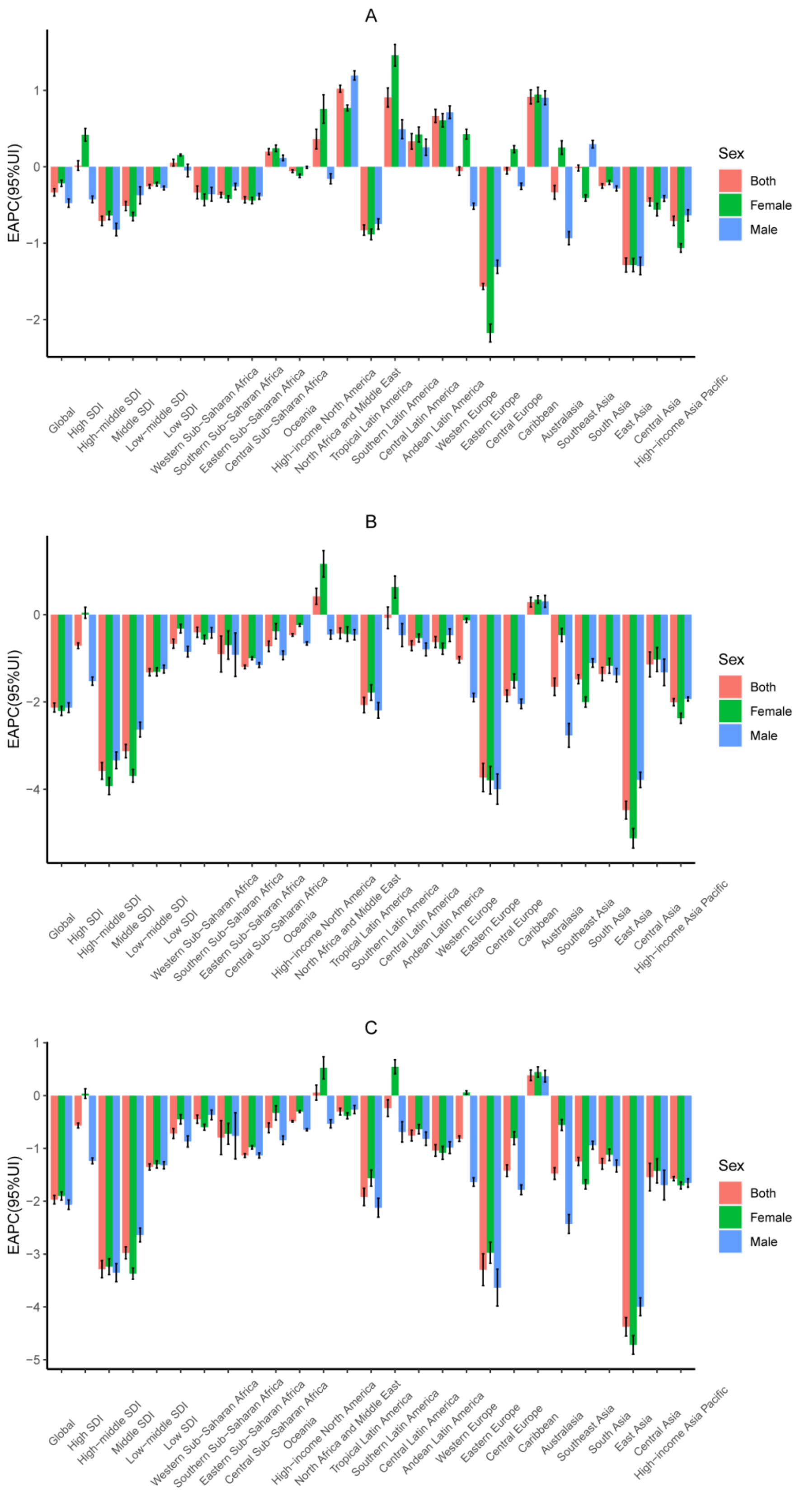

Supplementary Figure S3 The correlation between EAPC and Chronic Obstructive Pulmonary Disease ASR (incidence (a), death (c), and DALY (e)) in 1990 and HDI (incidence (b), death (d), and DALY (f)) in 2019.

Note: The circles represent countries that were available on SDI data. The size of circle represents the number of Chronic Obstructive Pulmonary Disease patients. The ρ indices Pearson's correlation coefficient and p values were derived from Pearson's correlation analysis. ASR, age-standardized incidence/death/DALY rate; EAPC, estimated annual percentage change; SDI, socio-demographic index

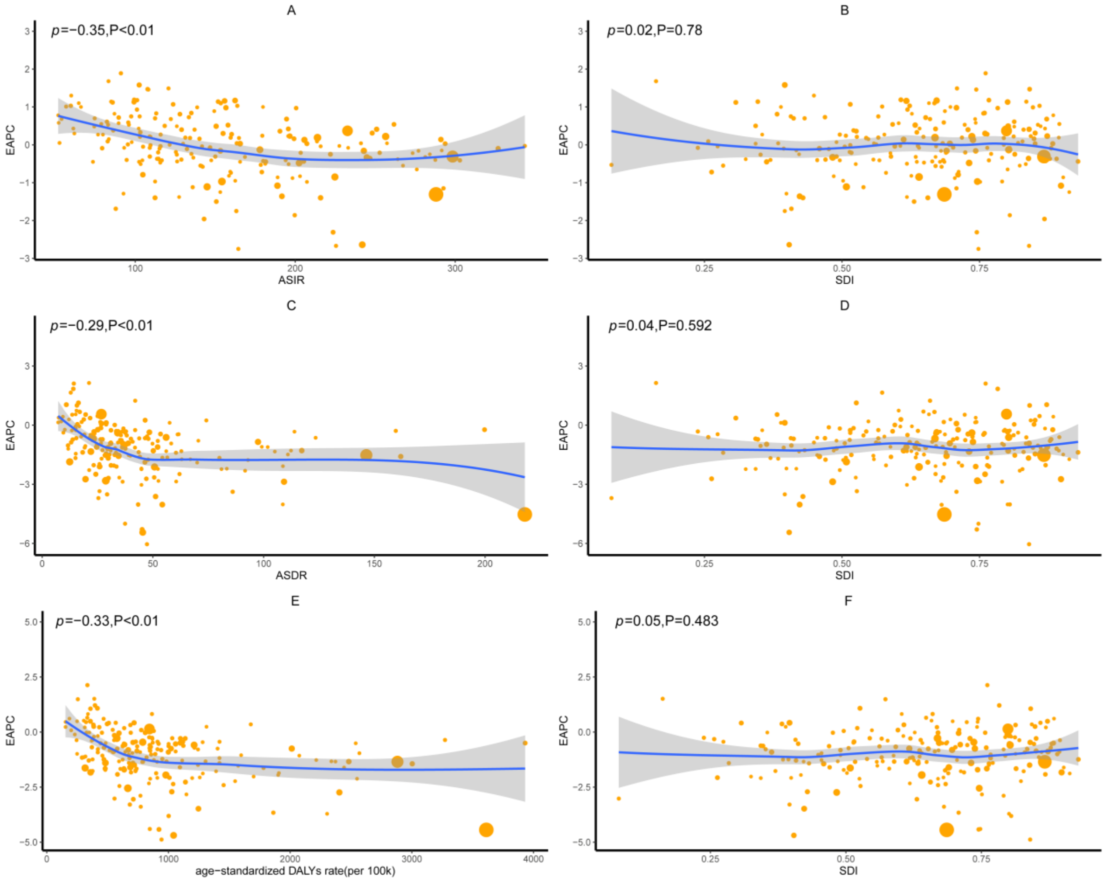

Supplementary Figure S4 The proportion of different ages in chronic obstructive pulmonary disease incidence (A) and death (B) by years.

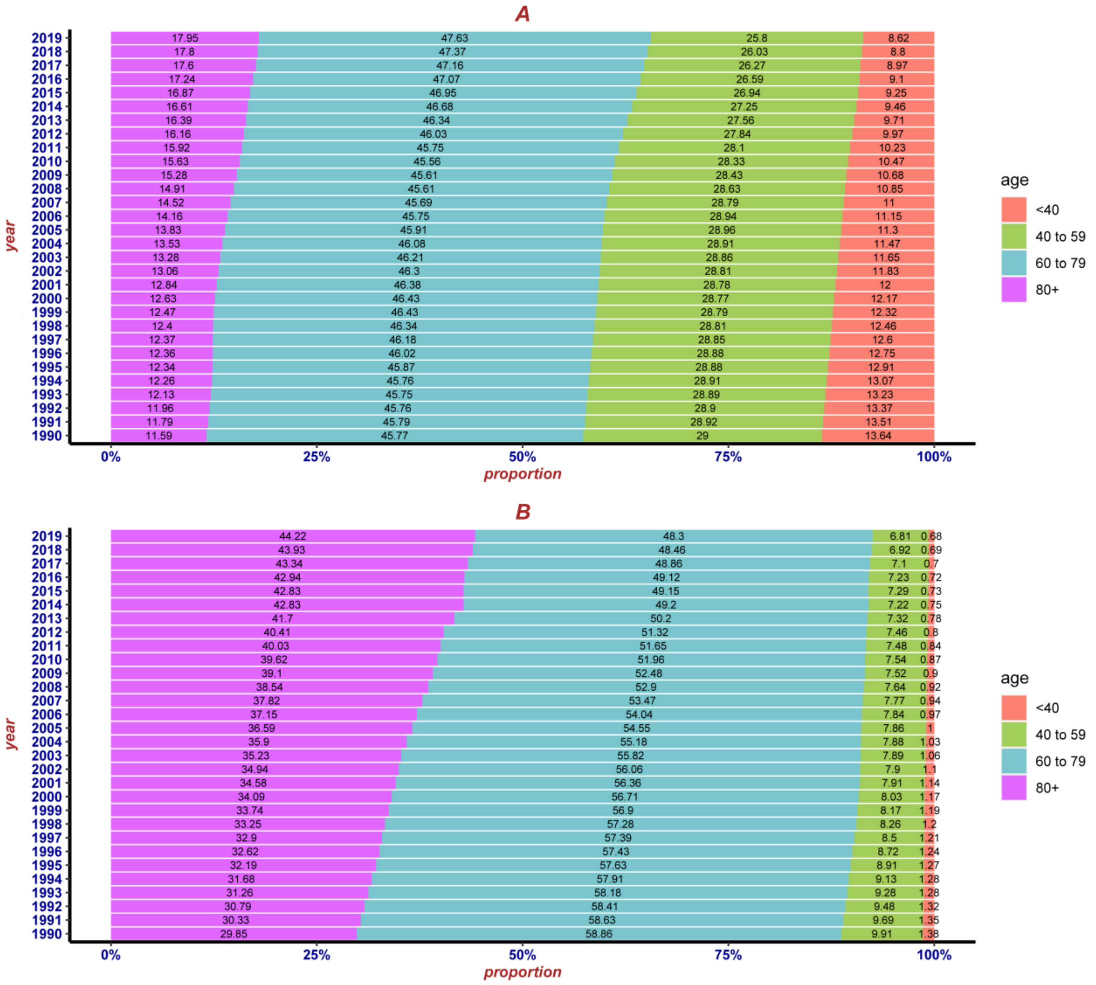

Supplementary Figure S5 The global EAPC of chronic obstructive pulmonary disease for both sexes in 204 countries.

Note: A: The EAPC of ASIR; B: The EAPC of ASDR; C: The EAPC of age-standardized DALY rate.

Supplementary Figure S5A: The EAPC of ASIR

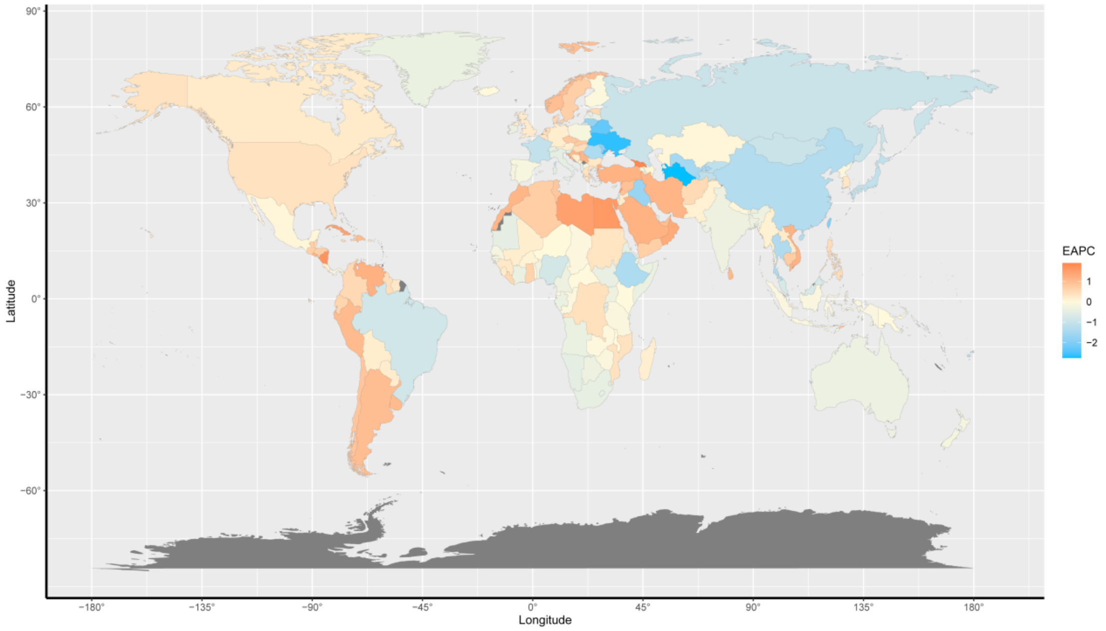

Supplementary Figure S5B: The EAPC of ASDR

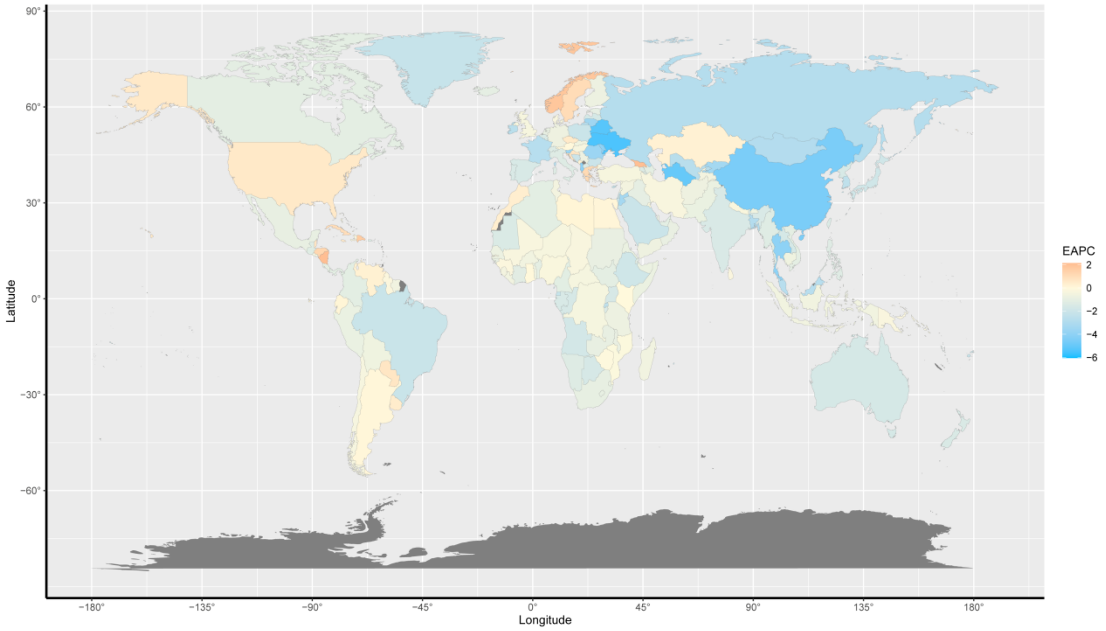

Supplementary Figure S5C: The EAPC of age-standardized DALY rate

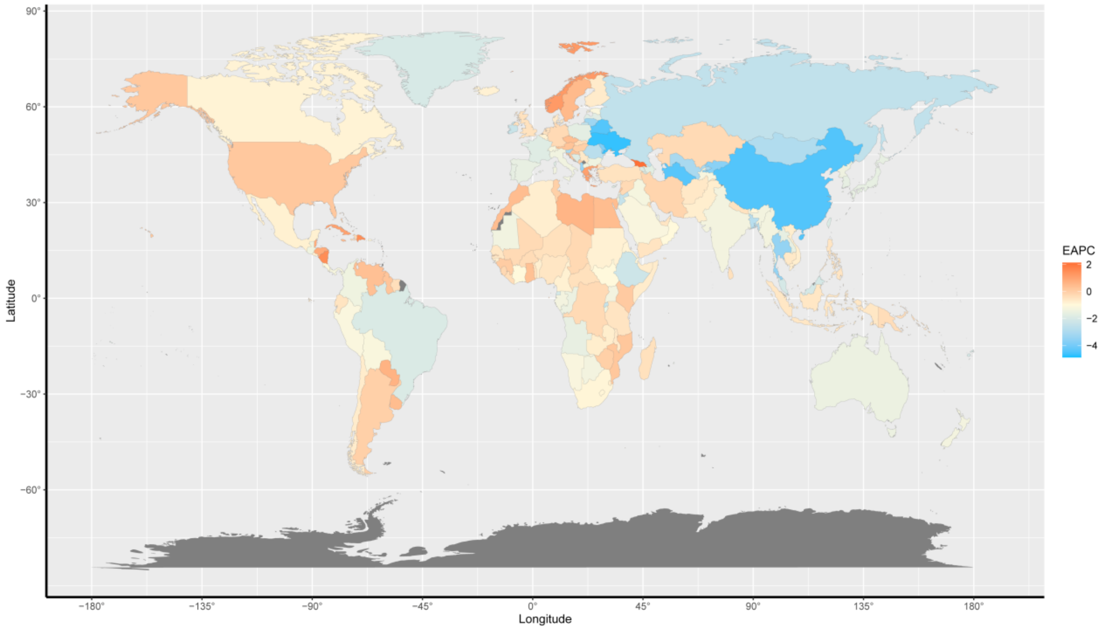

Supplementary Figure S6 The global EAPC of chronic obstructive pulmonary disease for Female in 204 countries.

Note: A: The EAPC of ASIR; B: The EAPC of ASDR; C: The EAPC of age-standardized DALY rate.

Supplementary Figure S6A: The EAPC of ASIR

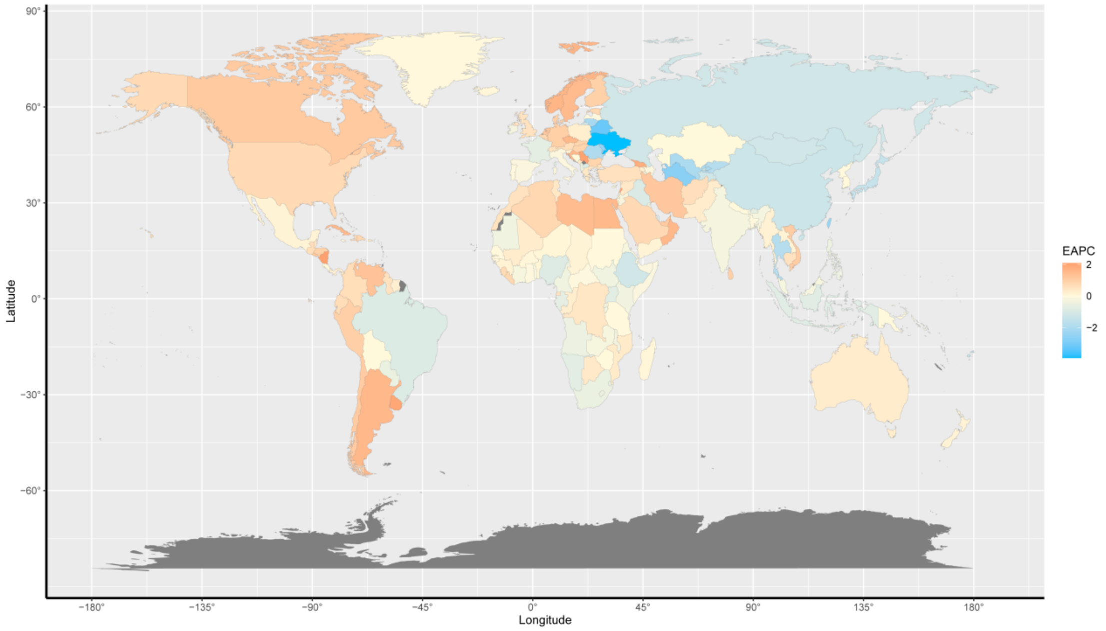

Supplementary Figure S6B: The EAPC of ASDR

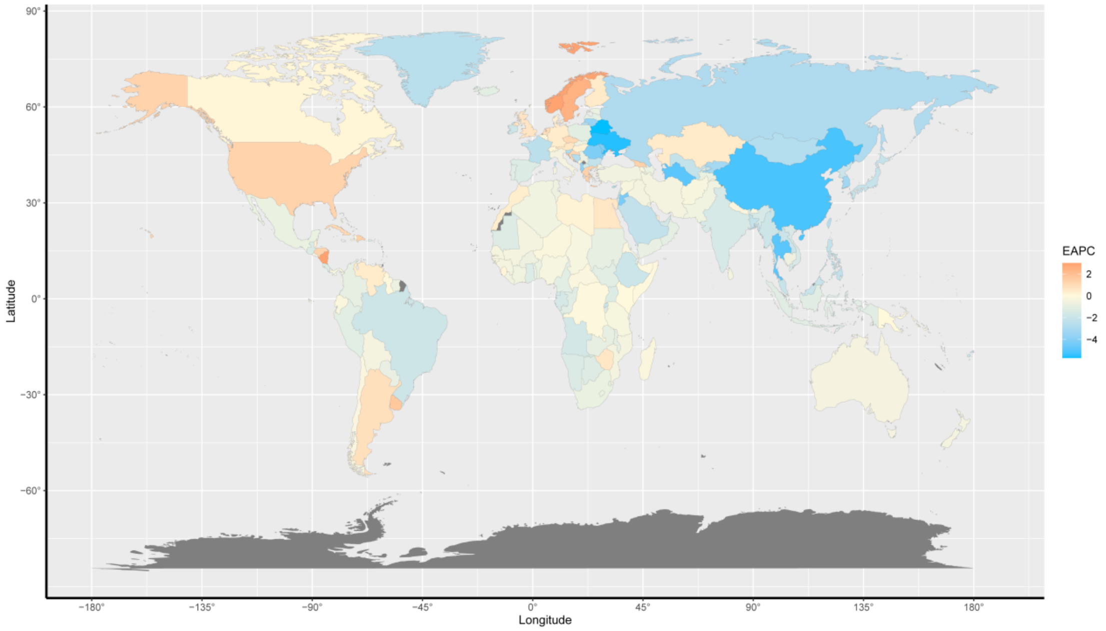

Supplementary Figure S6C: The EAPC of age-standardized DALY rate

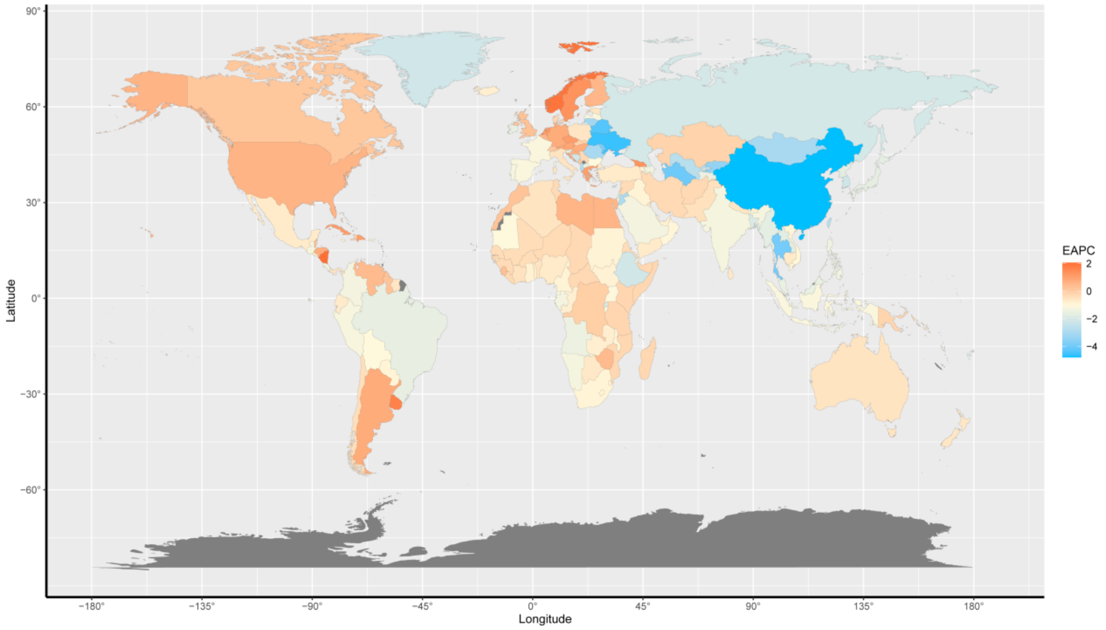

Supplementary Figure S7 The global EAPC of chronic obstructive pulmonary disease for male in 204 countries.

Note: A: The EAPC of ASIR; B: The EAPC of ASDR; C: The EAPC of age-standardized DALY rate.

Supplementary Figure S7A: The EAPC of ASIR

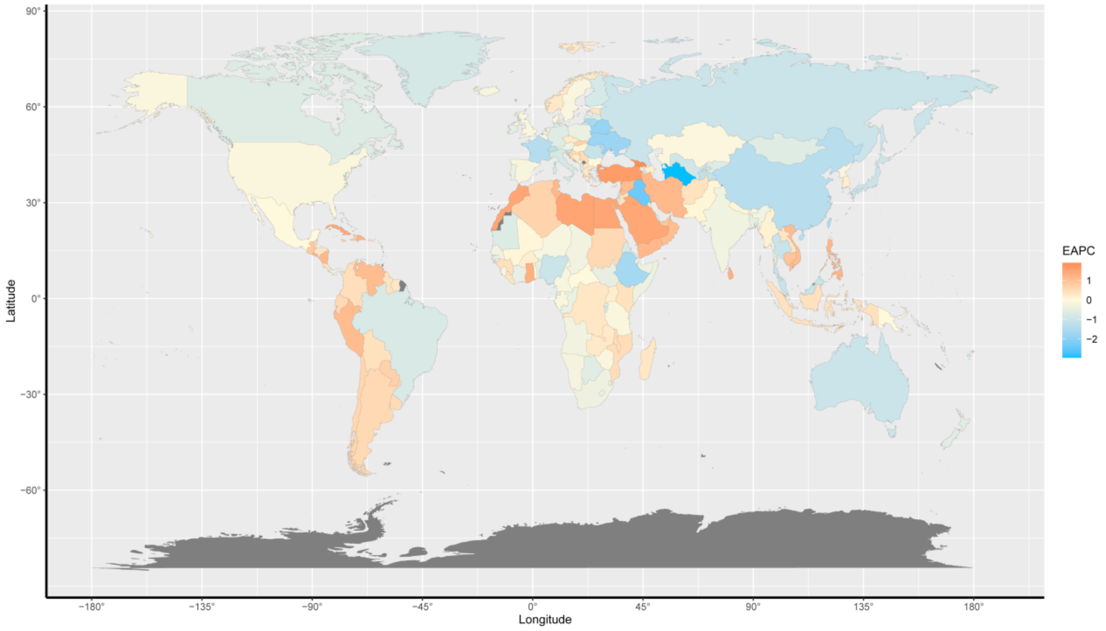

Supplementary Figure S7B: The EAPC of ASDR

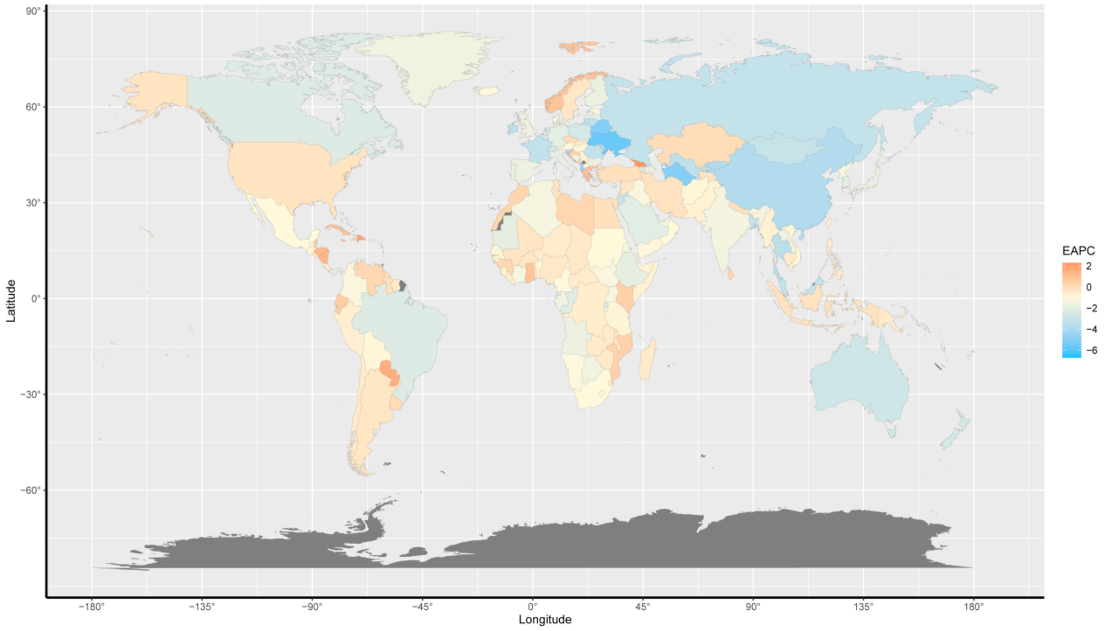

Supplementary Figure S7C: The EAPC of age-standardized DALY rate

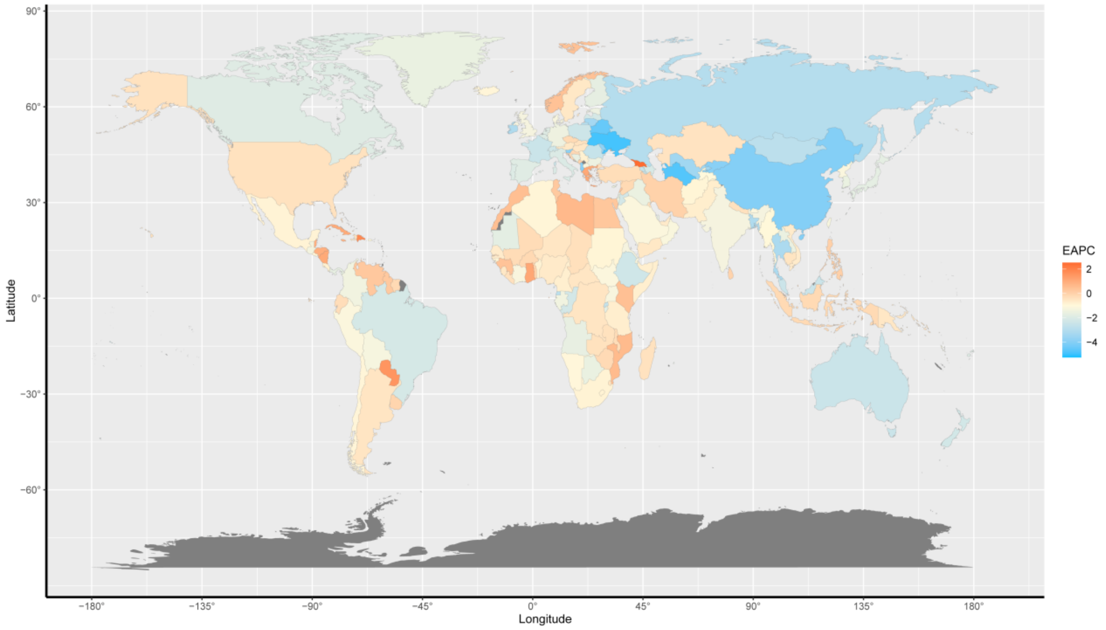

Supplementary Figure S8 The global disease burden of chronic obstructive pulmonary disease for Female in 204 countries.

Note: A: The ASIR of chronic obstructive pulmonary disease in 2019; B: The ASDR of chronic obstructive pulmonary disease in 2019; C: The age-standardized DALY rate of chronic obstructive pulmonary disease in 2019. ASIR: age-standardized incidence rate; ASDR: age-standardized death rate.

Supplementary Figure S8A: The ASIR of chronic obstructive pulmonary disease in 2019

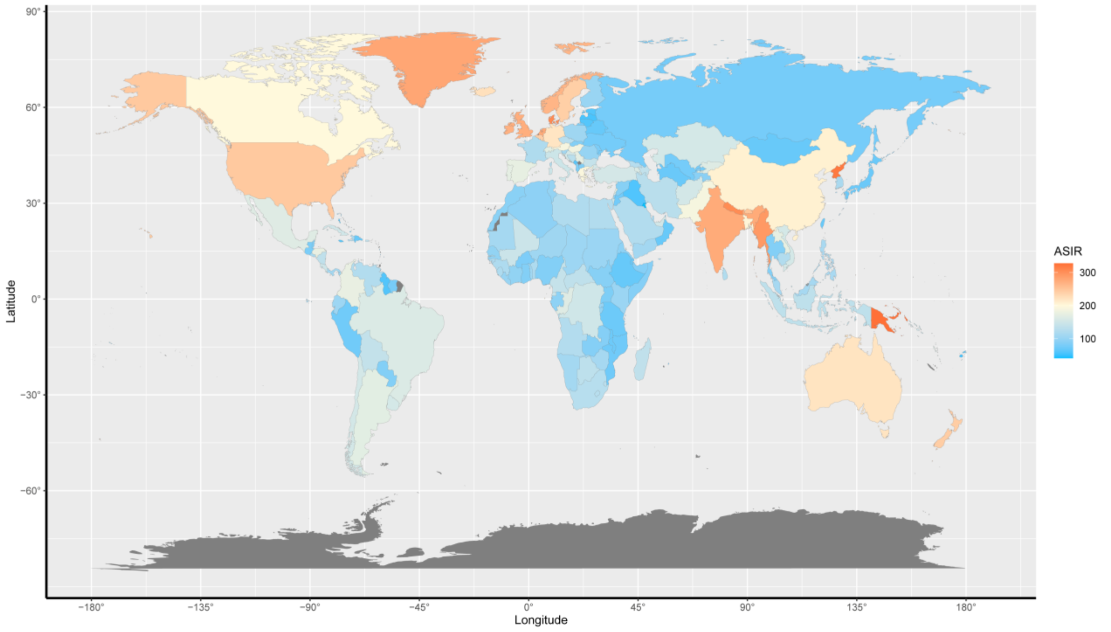

Supplementary Figure S8B: The ASDR of chronic obstructive pulmonary disease in 2019

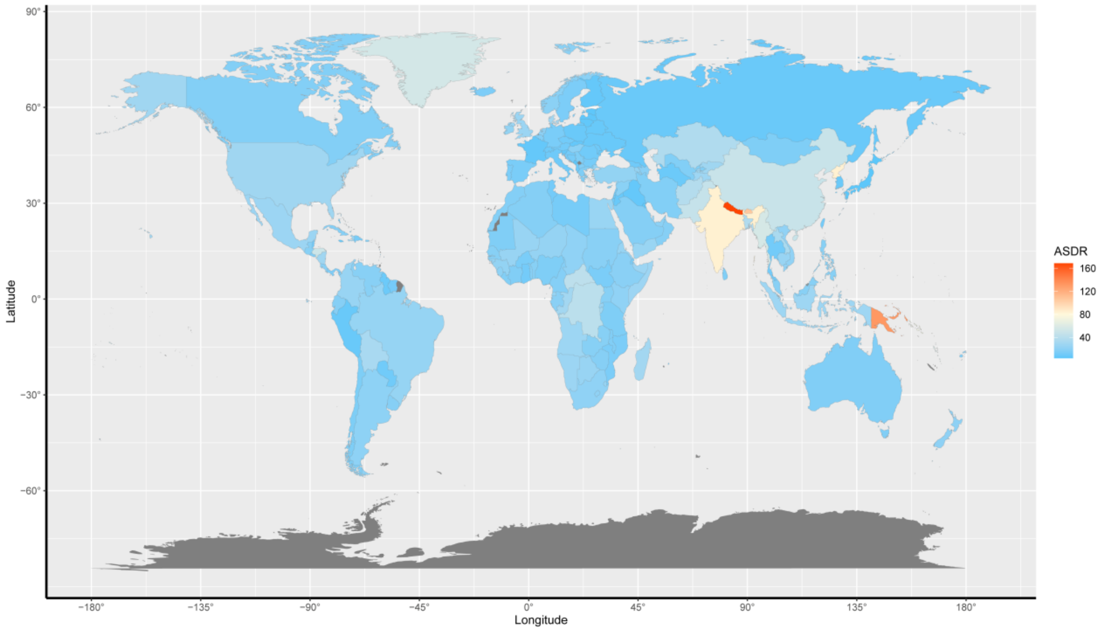

Supplementary Figure S8C: The age-standardized DALY rate of chronic obstructive pulmonary disease in 2019

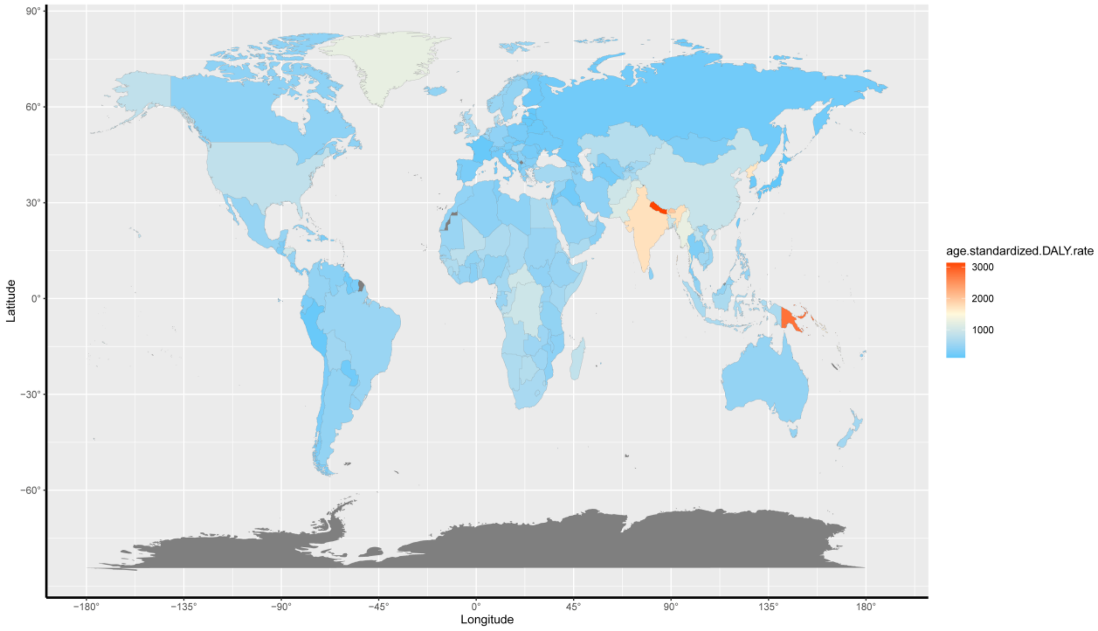

Supplementary Figure S9 The global disease burden of chronic obstructive pulmonary disease for male in 204 countries.

Note: A: The ASIR of Chronic Obstructive Pulmonary Disease in 2019; B: The ASDR of chronic obstructive pulmonary disease in 2019; C: The age-standardized DALY rate of chronic obstructive pulmonary disease in 2019. ASIR: age-standardized incidence rate; ASDR: age-standardized death rate.

Supplementary Figure S9A: The ASIR of chronic obstructive pulmonary disease in 2019

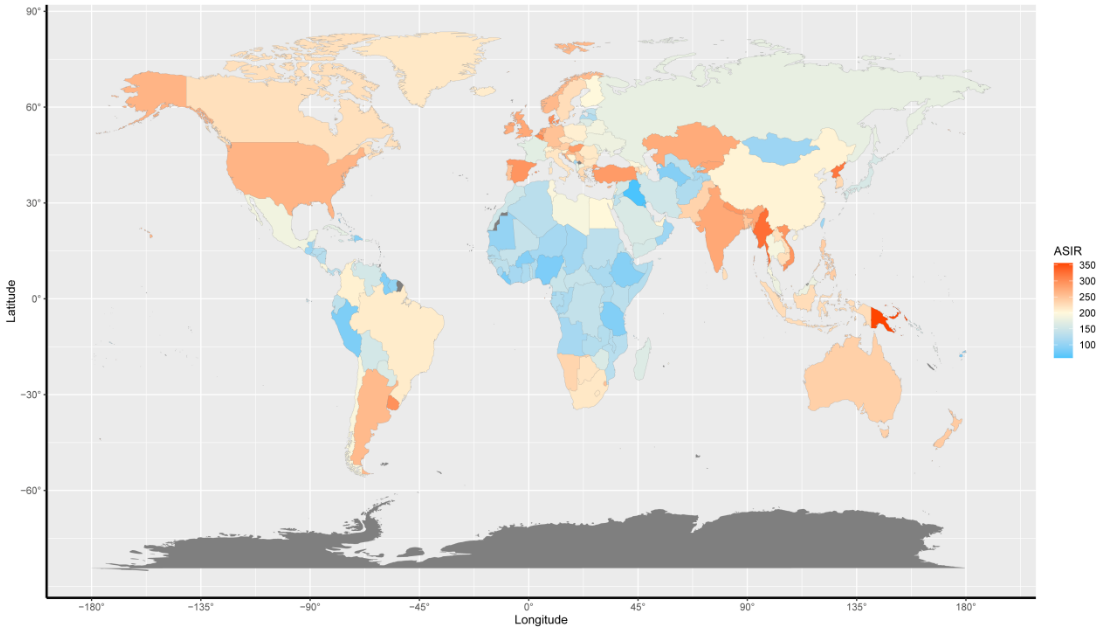

Supplementary Figure S9B: The ASDR of chronic obstructive pulmonary disease in 2019

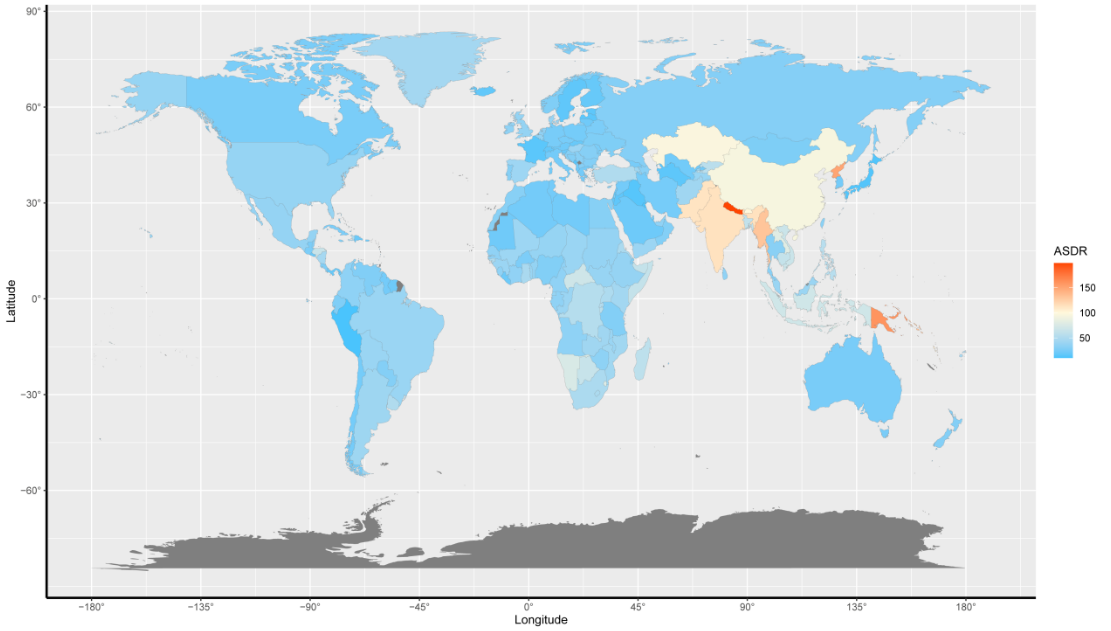

Supplementary Figure S9C: The age-standardized DALY rate of chronic obstructive pulmonary disease in 2019

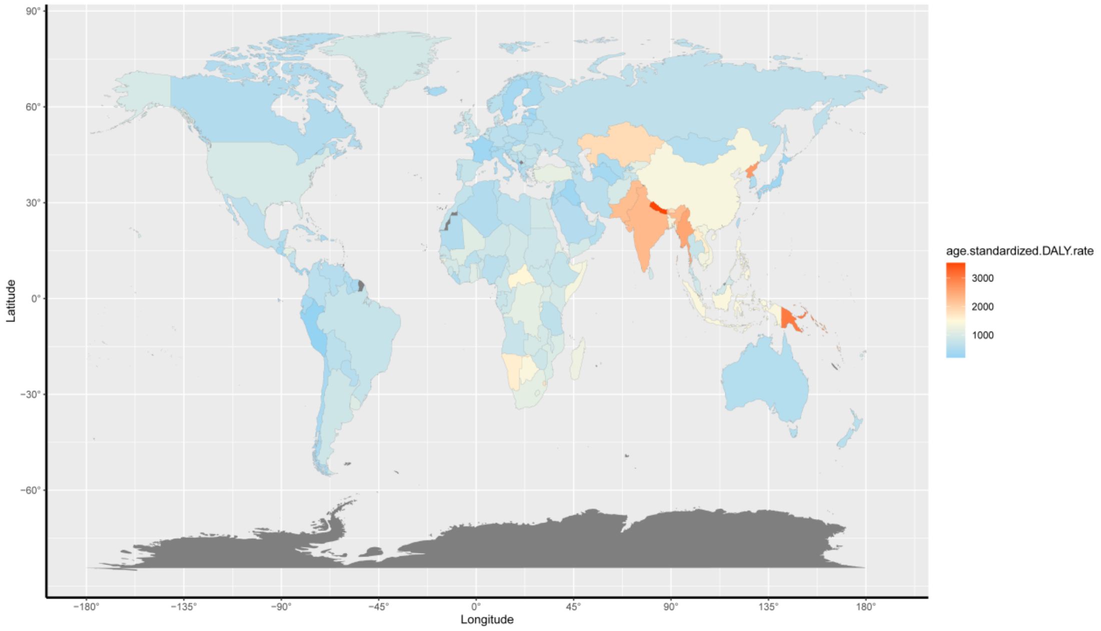

Supplementary Figure S10 Causes of deaths risk for Female in global (A), high SDI (B), high-middle SDI (C), middle SDI (D), middle-low SDI (E) and low SDI (F).

Note: SDI, socio-demographic index.

Supplementary Figure S10A: global

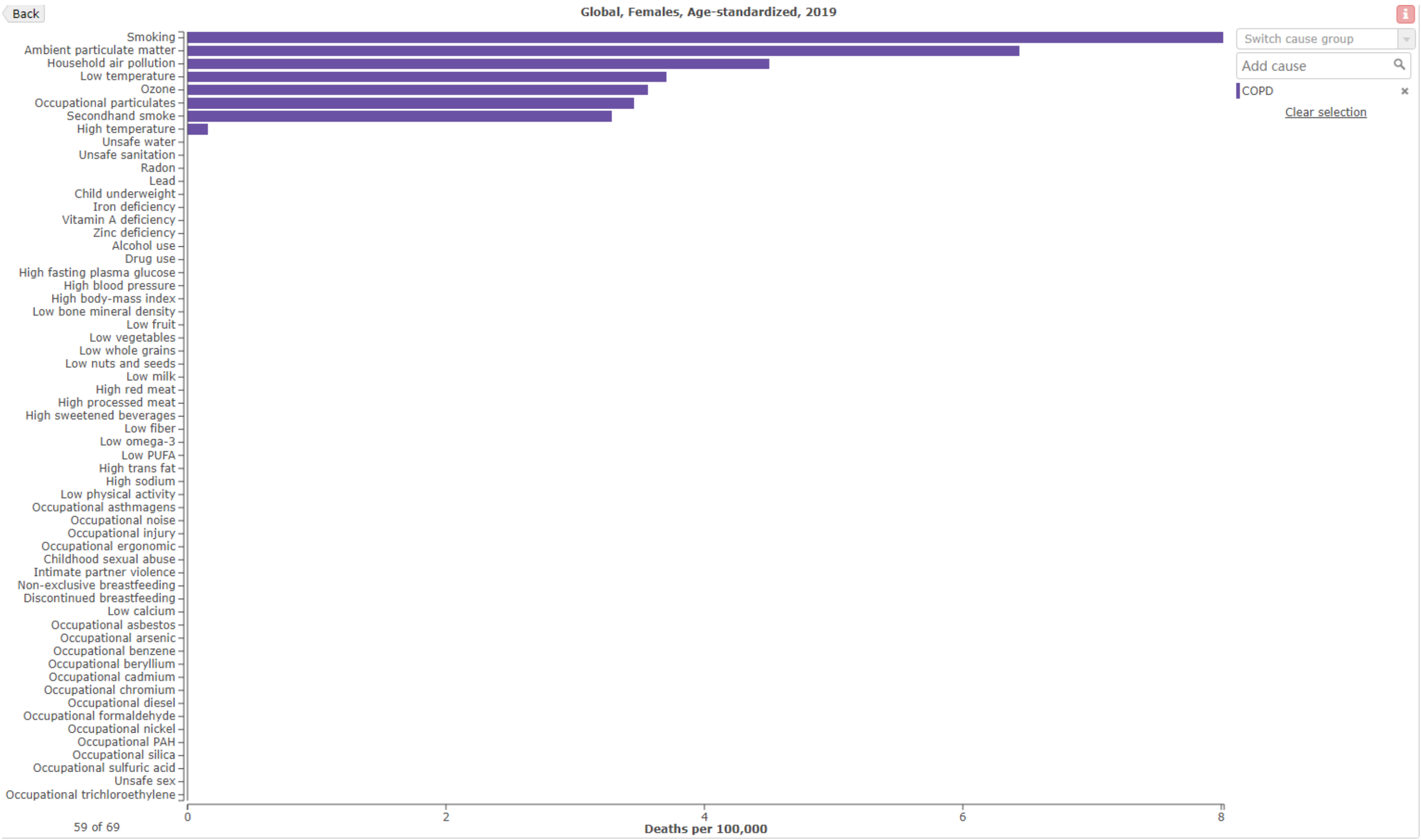

Supplementary Figure S10B: high SDI

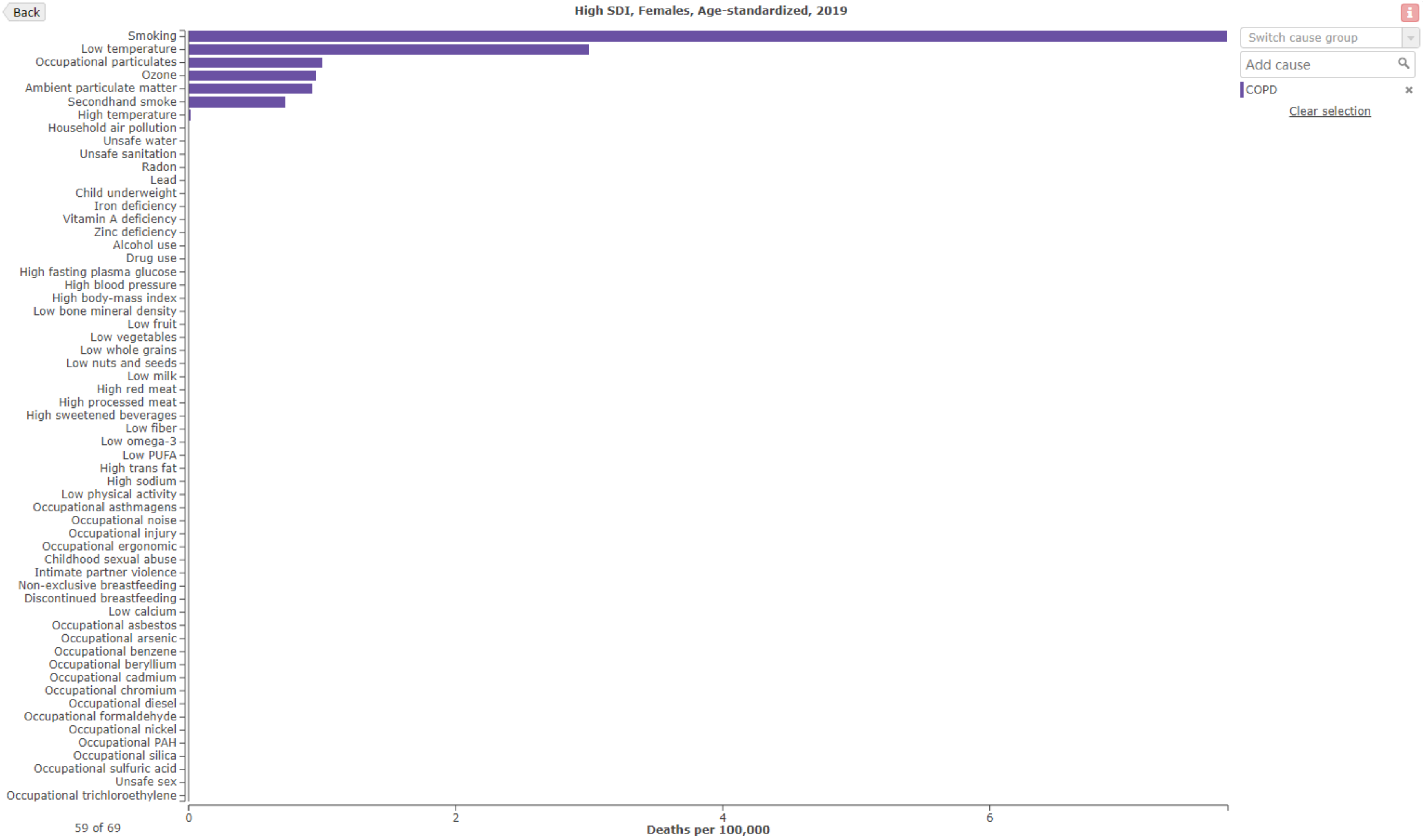

Supplementary Figure S10C: high-middle SDI

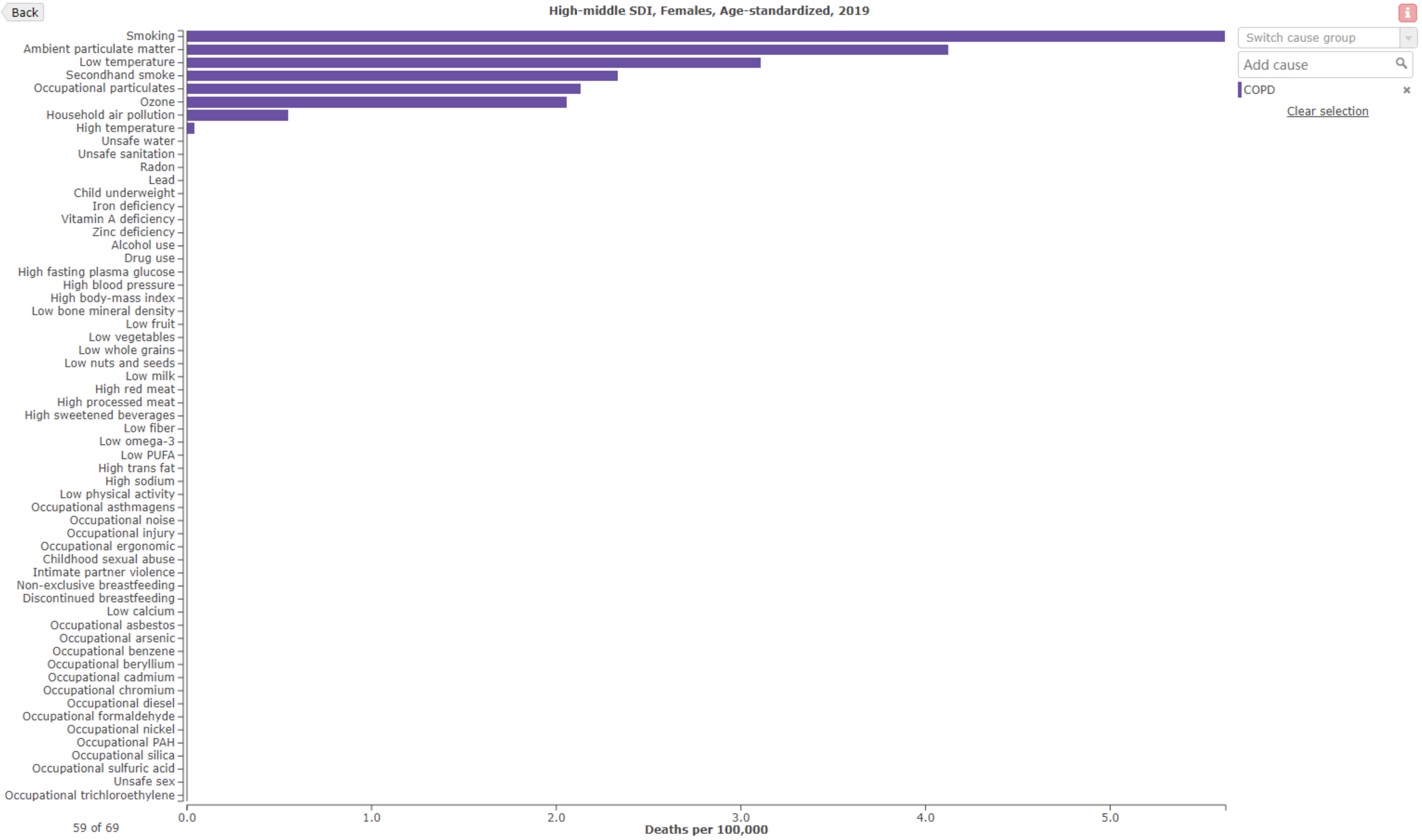

Supplementary Figure S10D: middle SDI

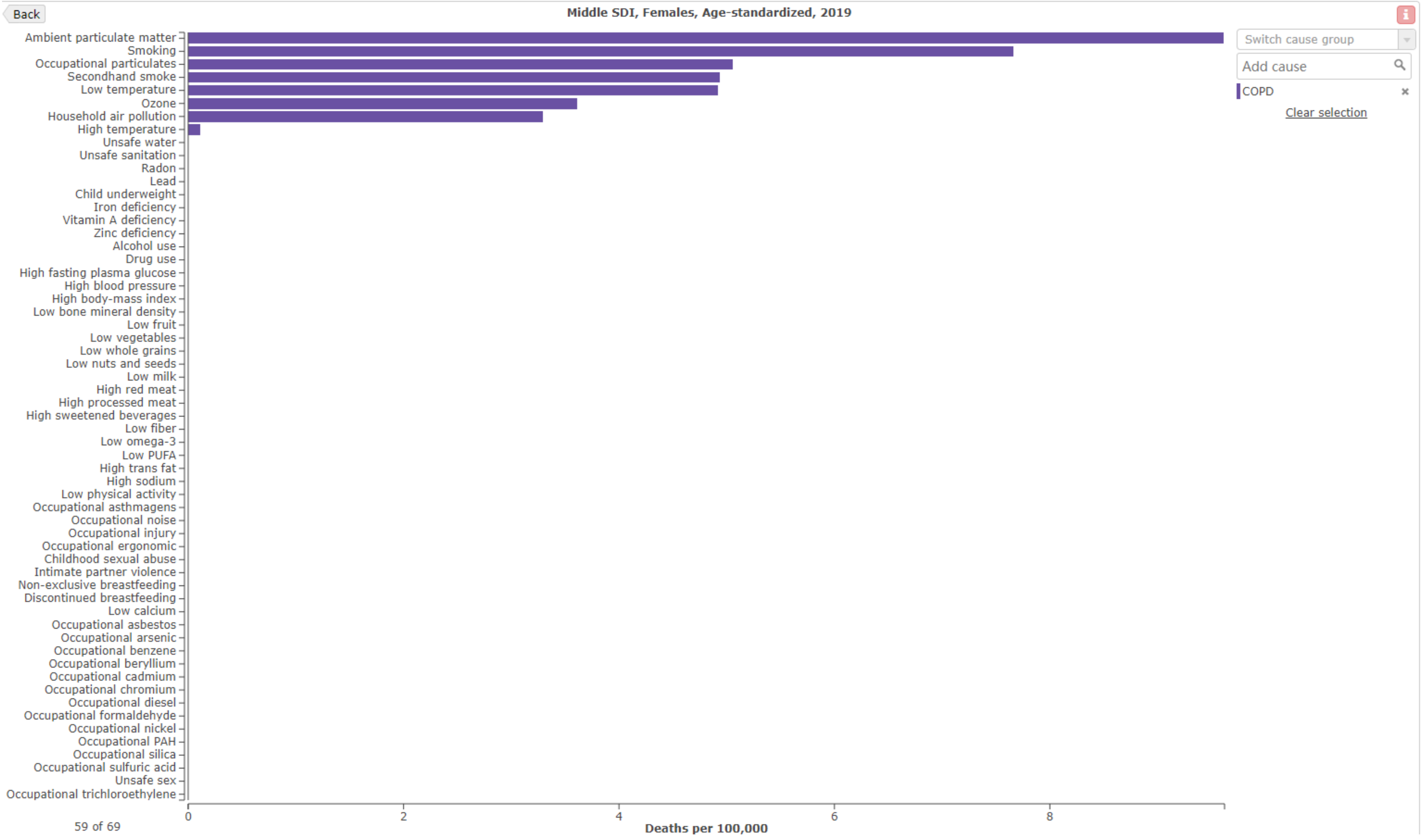

Supplementary Figure S10E: middle-low SDI

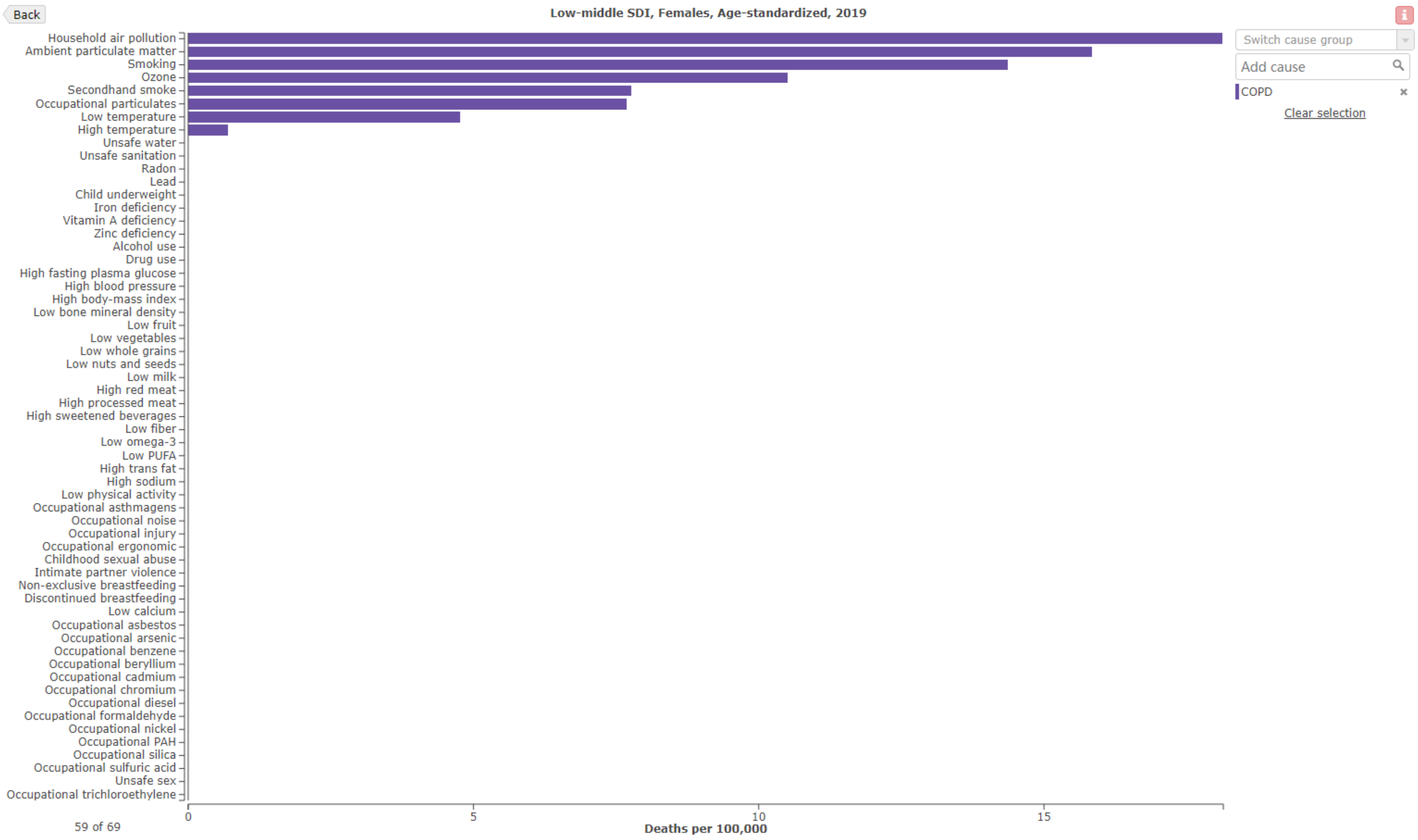

Supplementary Figure S10F: low SDI

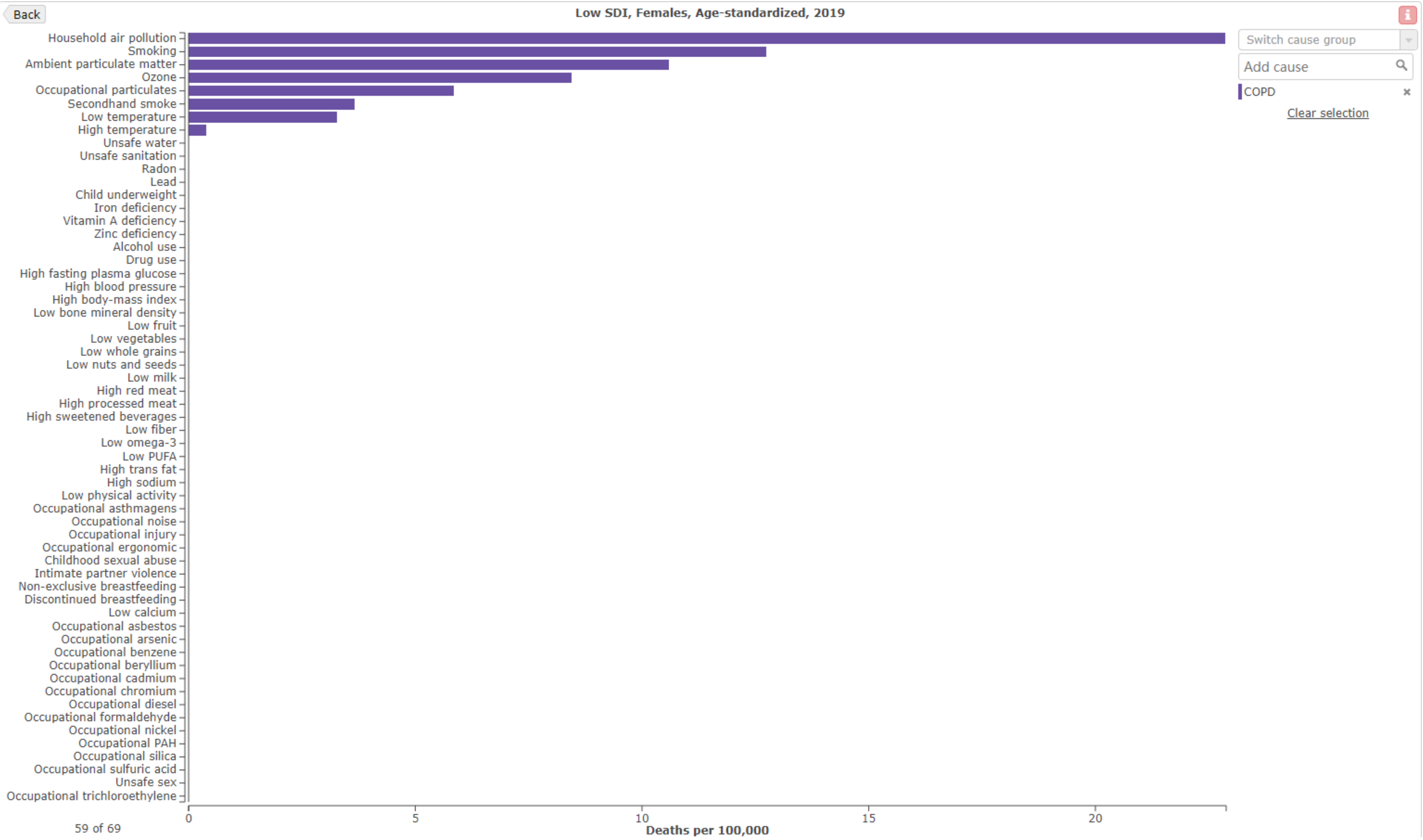

Supplementary Figure S11 Causes of deaths risk for male in global (A), high SDI (B), high-middle SDI (C), middle SDI (D), middle-low SDI (E) and low SDI (F).

Note: SDI, socio-demographic index.

Supplementary Figure S11A: global

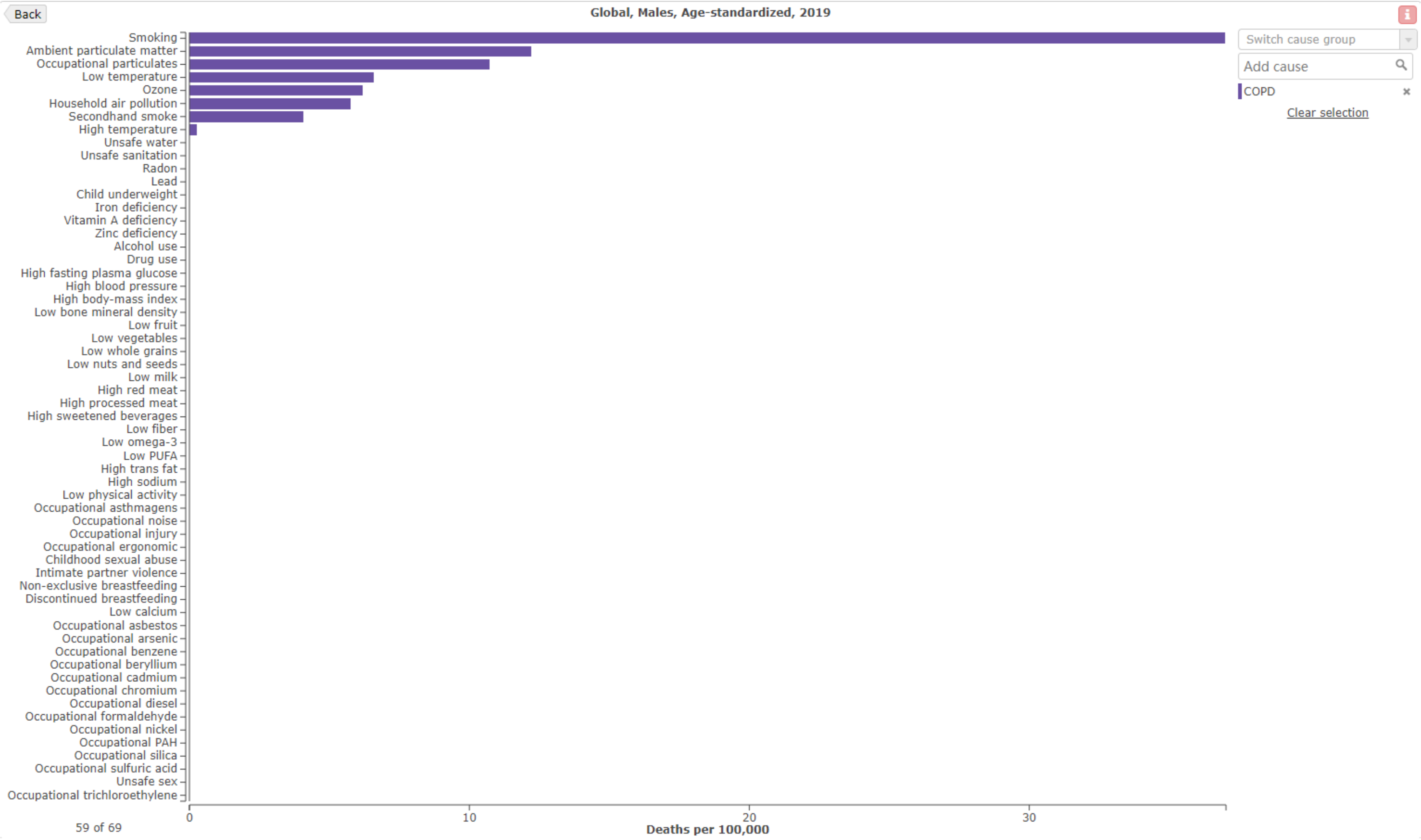

Supplementary Figure S11B: high SDI

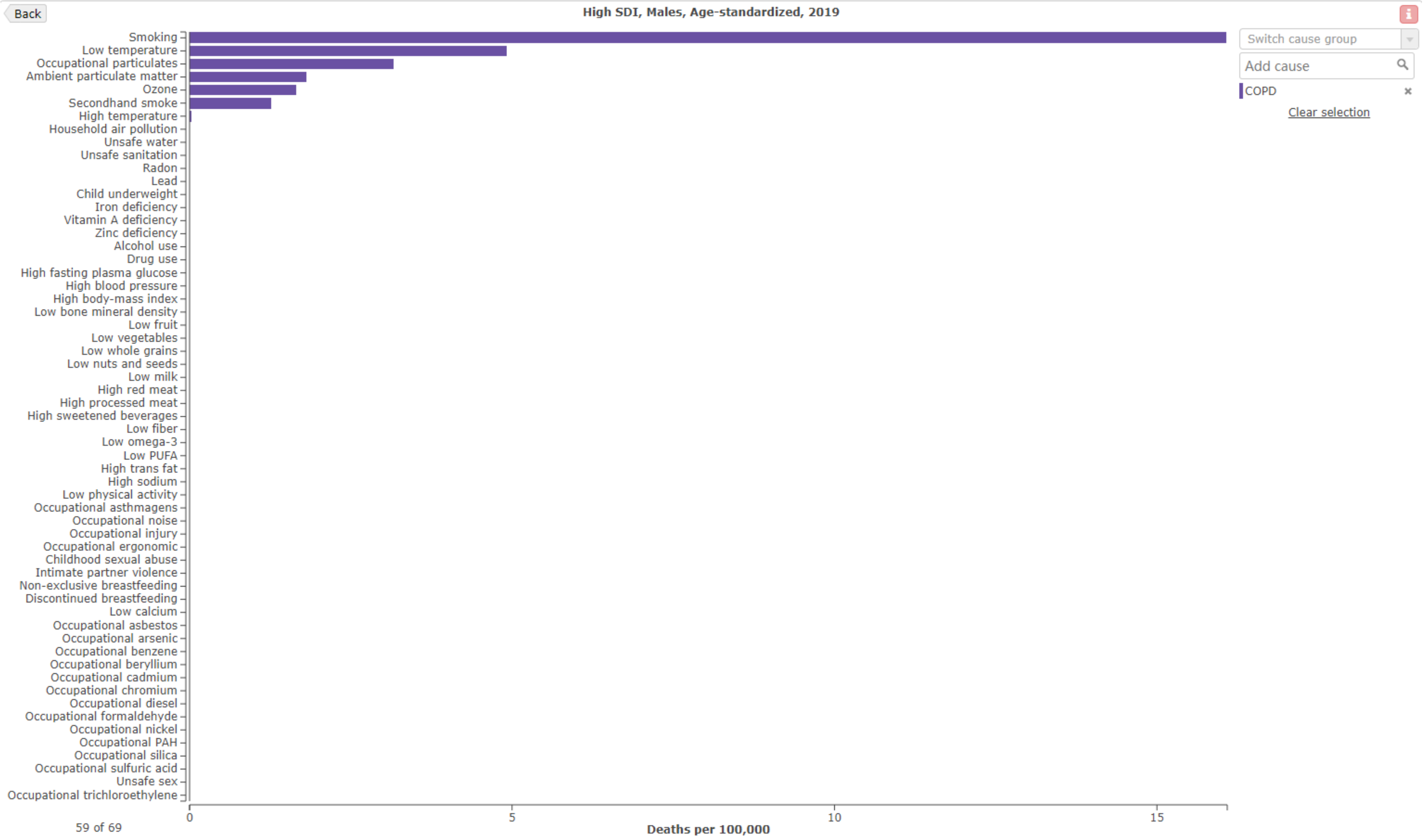

Supplementary Figure S11C: high-middle SDI

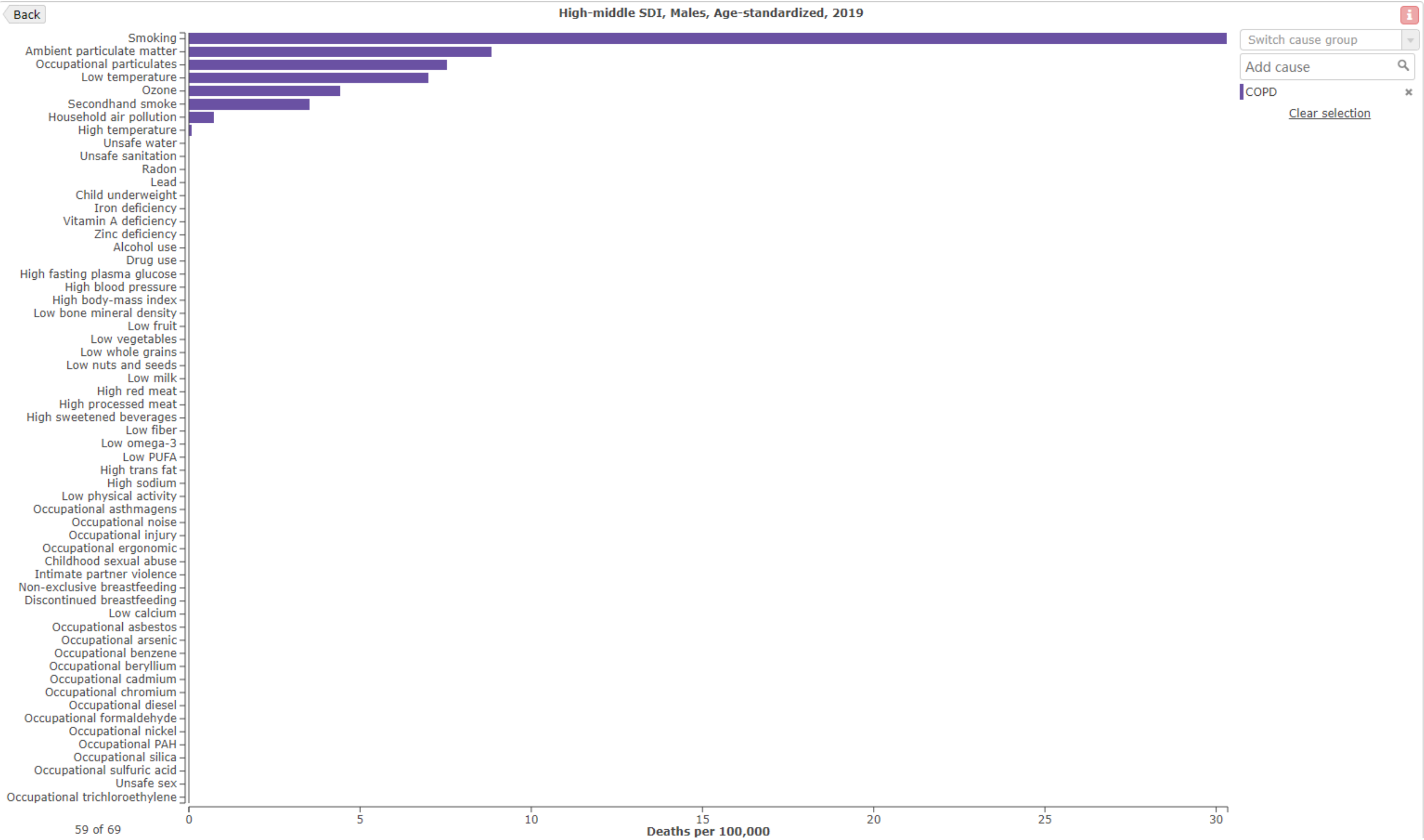

Supplementary Figure S11D: middle SDI

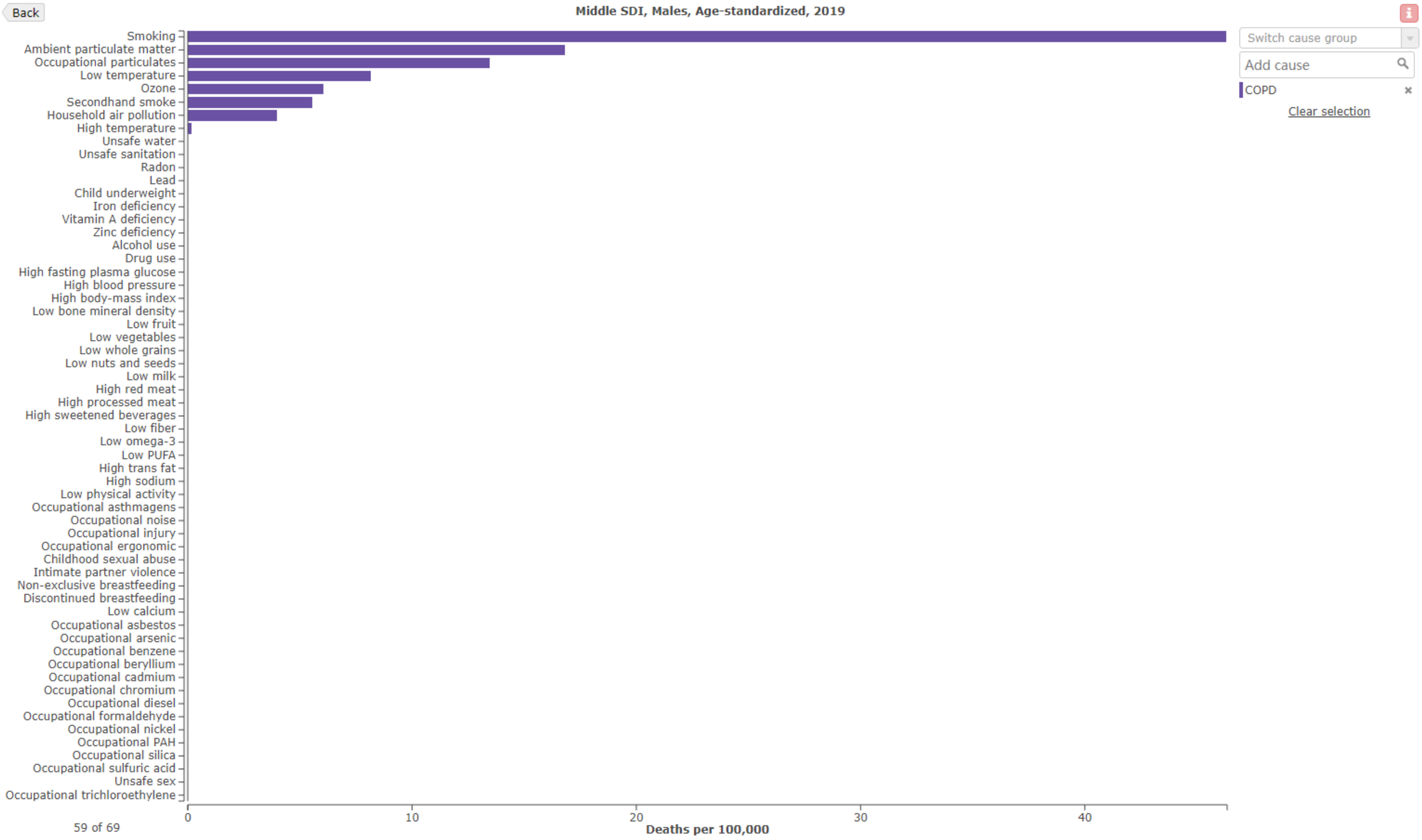

Supplementary Figure S11E: middle-low SDI

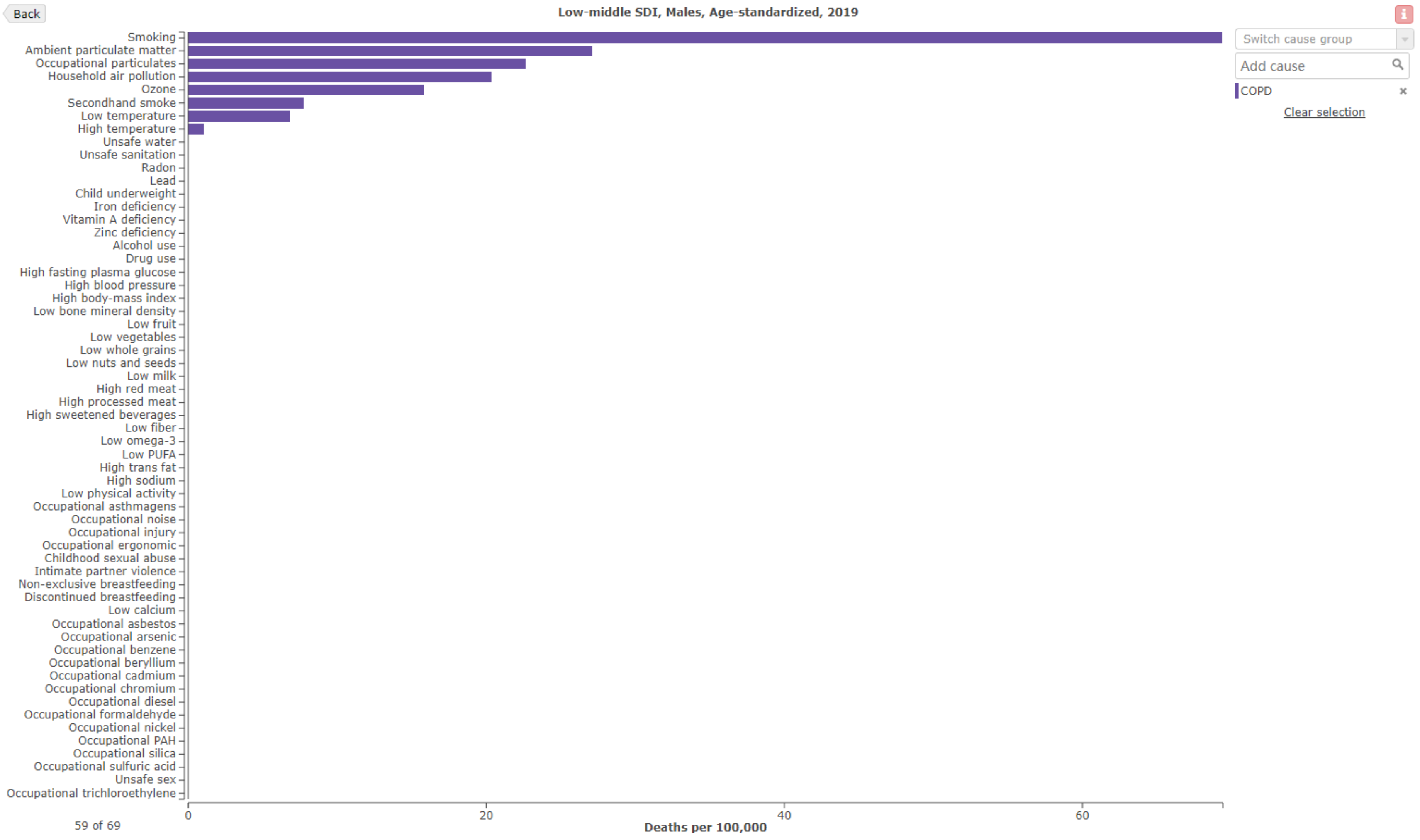

Supplementary Figure S11F: low SDI

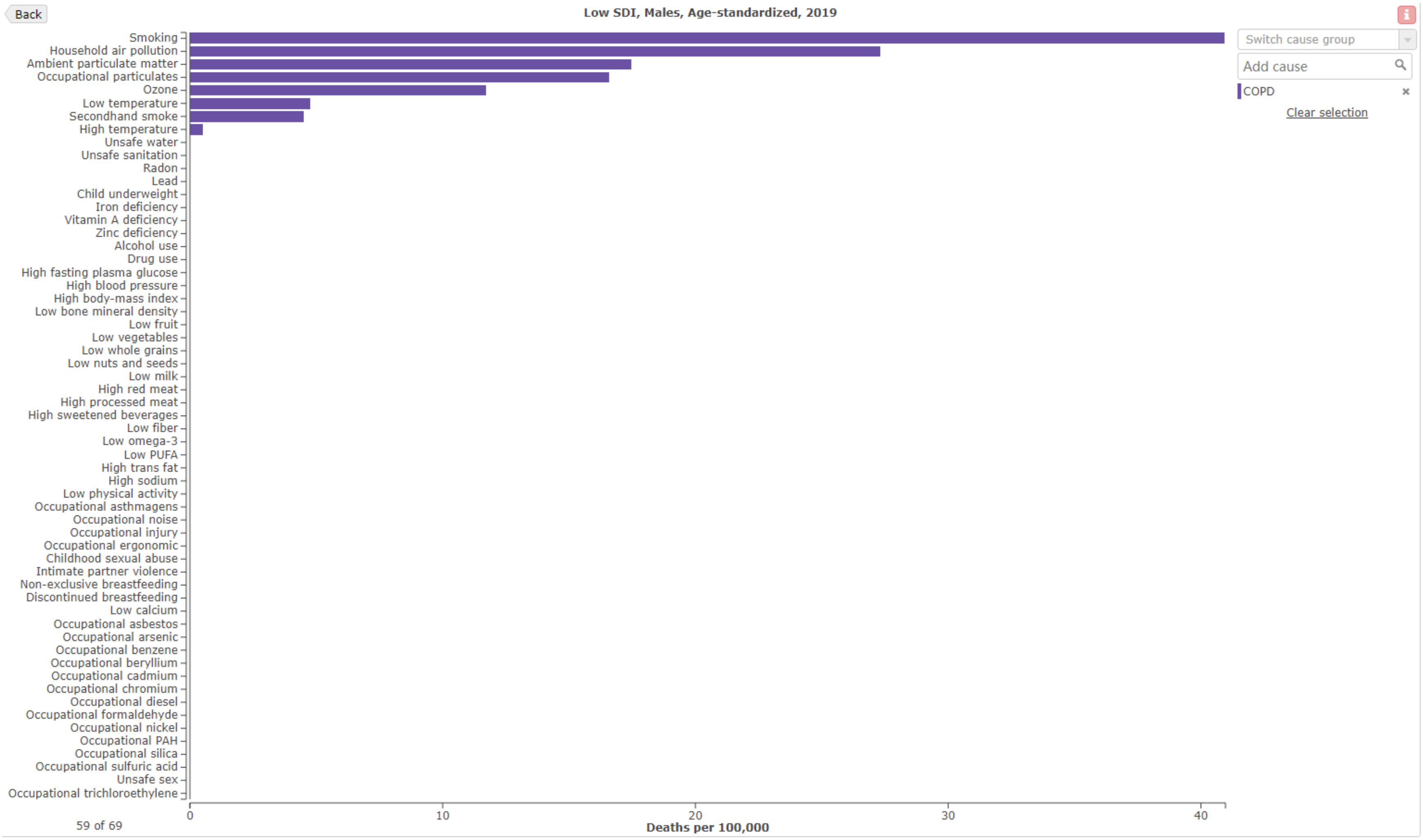

Supplementary Figure S12 The ratio of male to female ASDR among different age groups in global (A), high SDI (B), high-middle SDI (C), middle SDI (D), middle-low SDI (E) and low SDI (F).

Note: SDI, socio-demographic index. ASDR, age-standardized death rate.

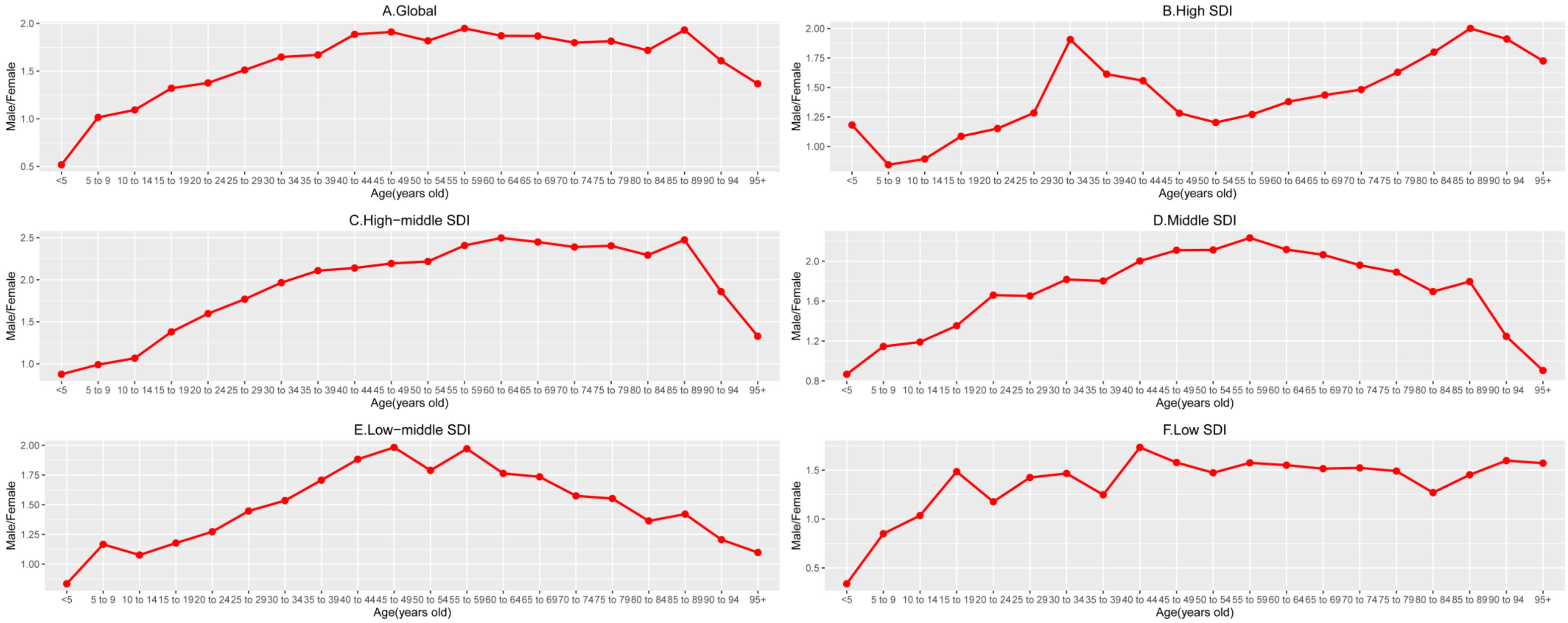

Supplementary Figure S13 Causes of DALYs risk for Female in global (A), high SDI (B), high-middle SDI (C), middle SDI (D), middle-low SDI (E) and low SDI (F).

Note: SDI, socio-demographic index.

Supplementary Figure S13A: global

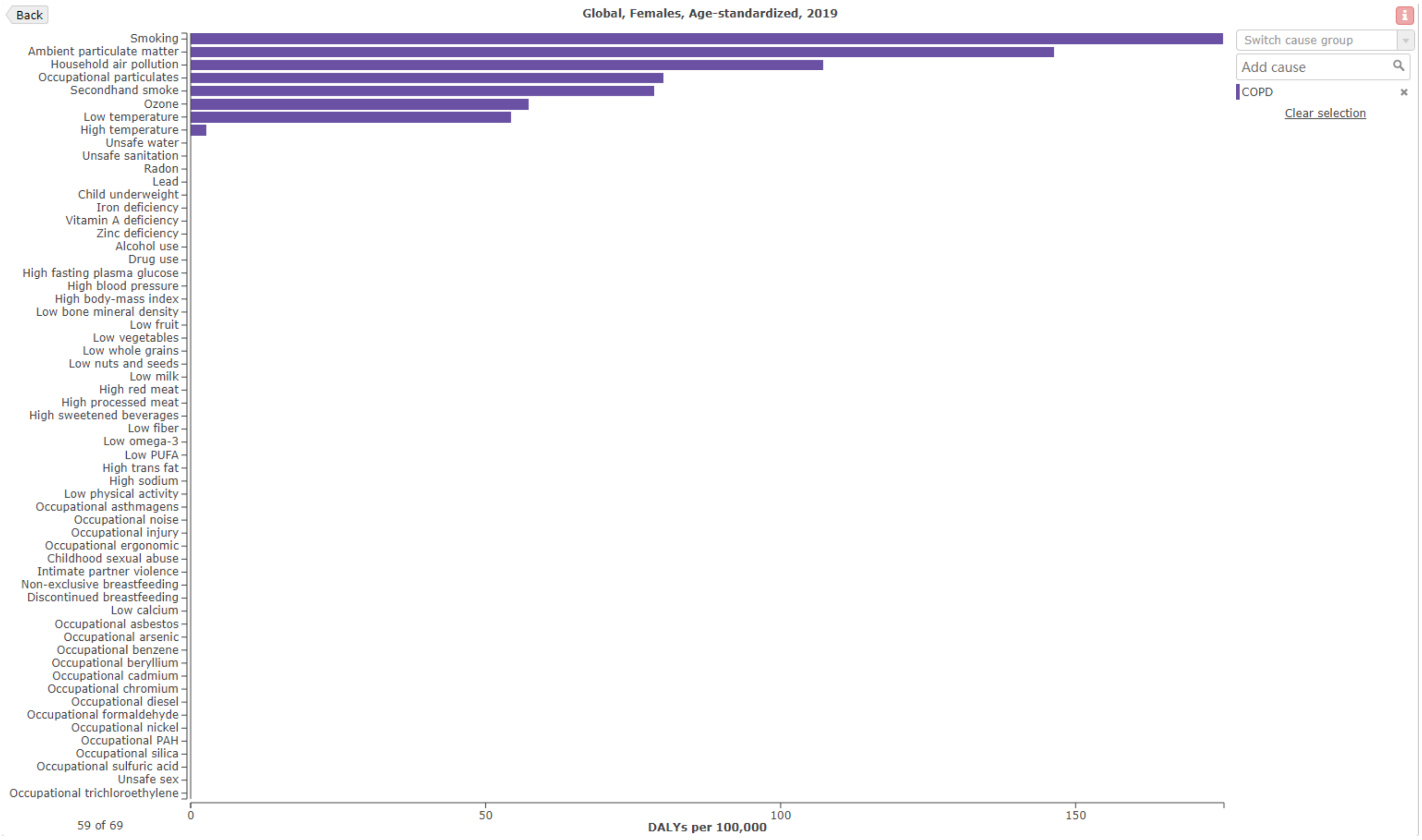

Supplementary Figure S13B: high SDI

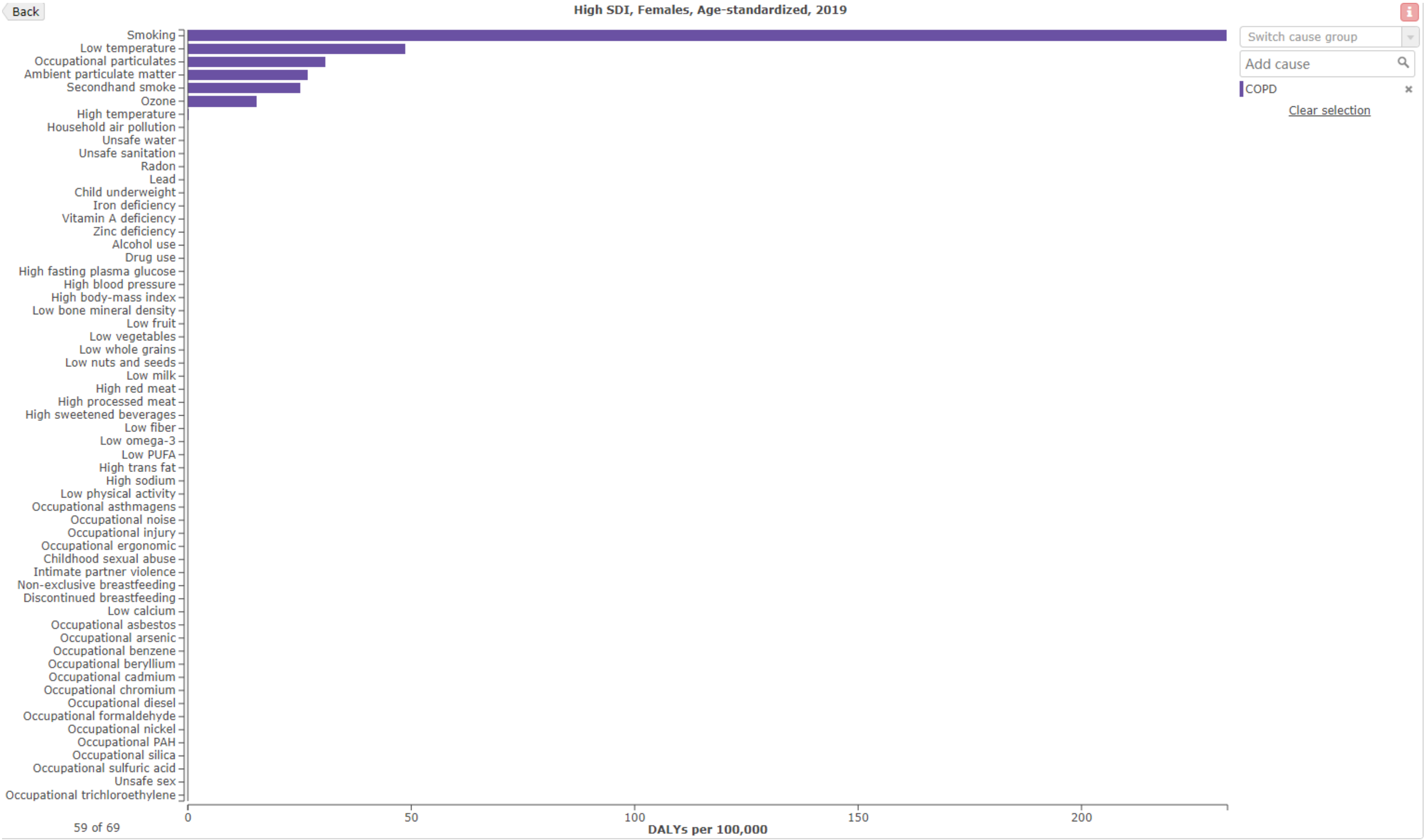

Supplementary Figure S13C: high-middle SDI

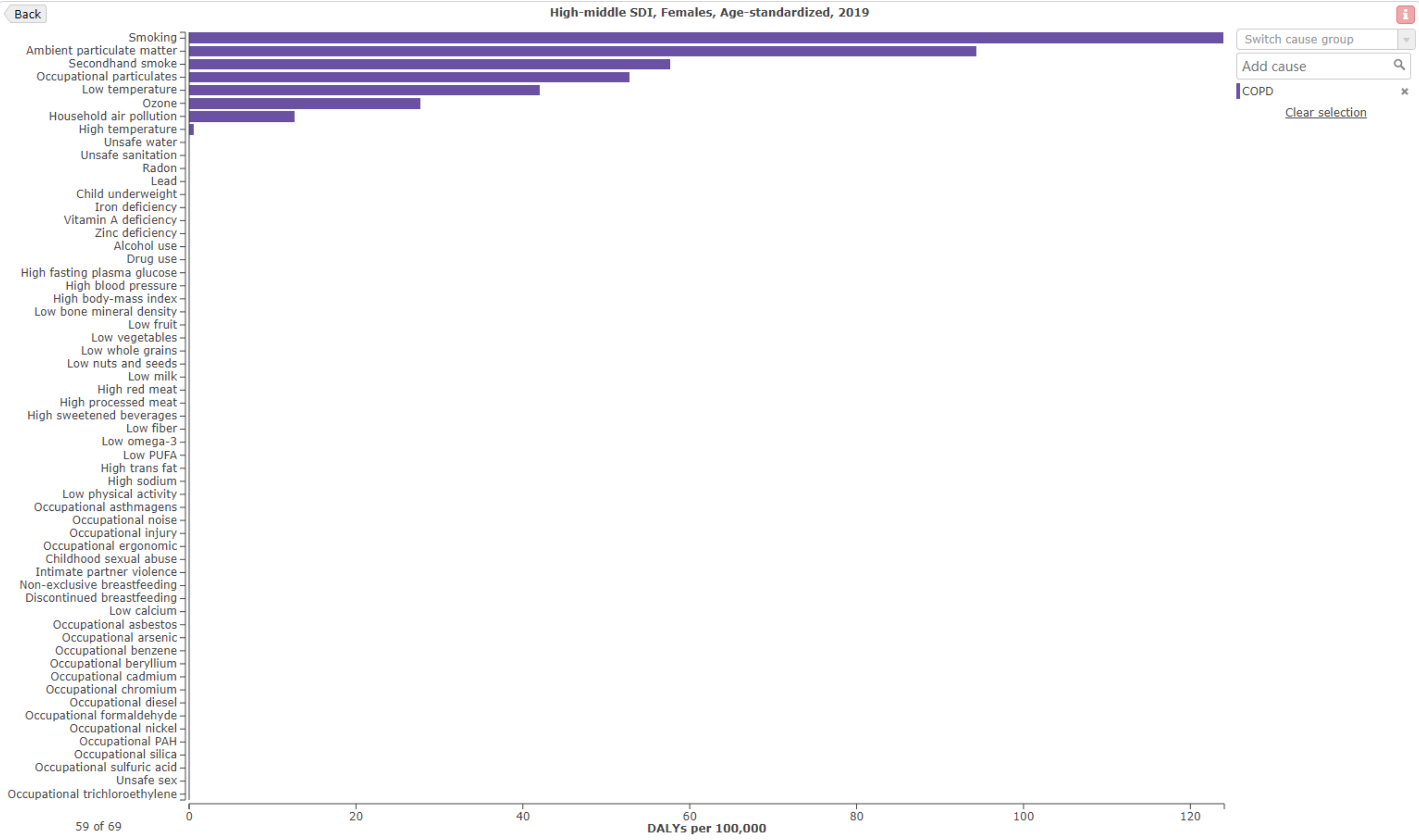

Supplementary Figure S13D: middle SDI

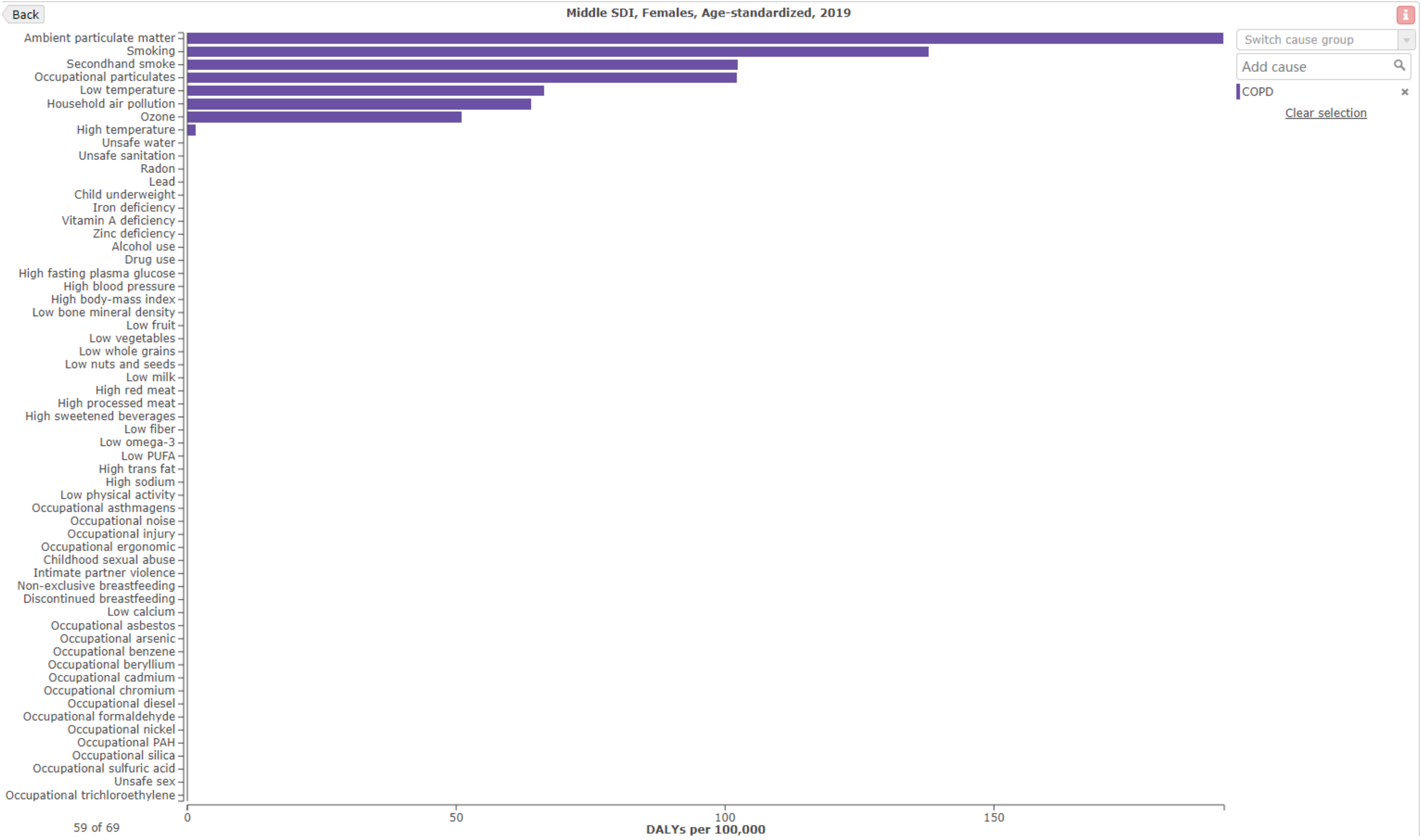

Supplementary Figure S13E: middle-low SDI

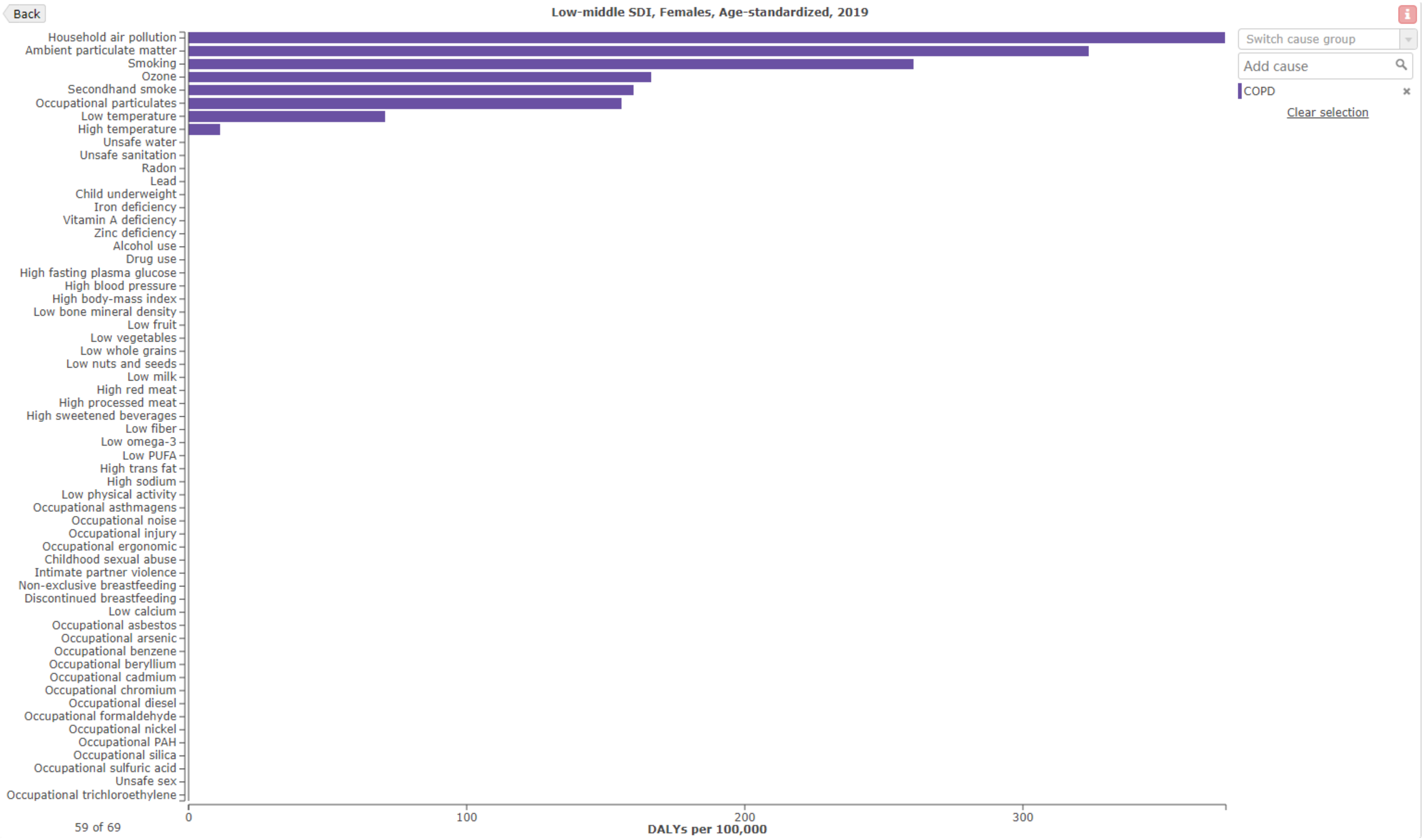

Supplementary Figure S13F: low SDI

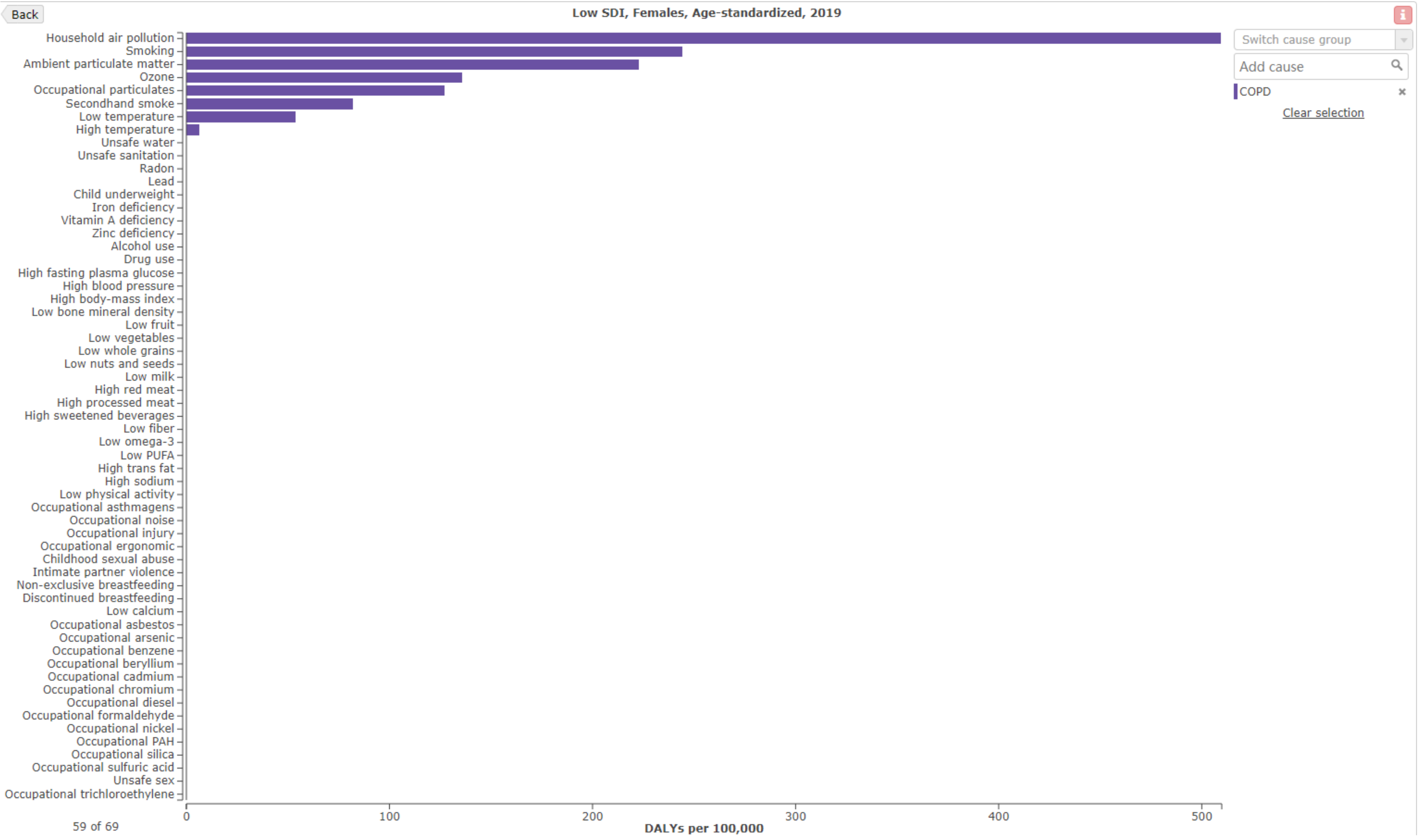

Supplementary Figure S14 Causes of DALYs risk for male in global (A), high SDI (B), high-middle SDI (C), middle SDI (D), middle-low SDI (E) and low SDI (F).

Note: SDI, socio-demographic index.

Supplementary Figure S14A: global

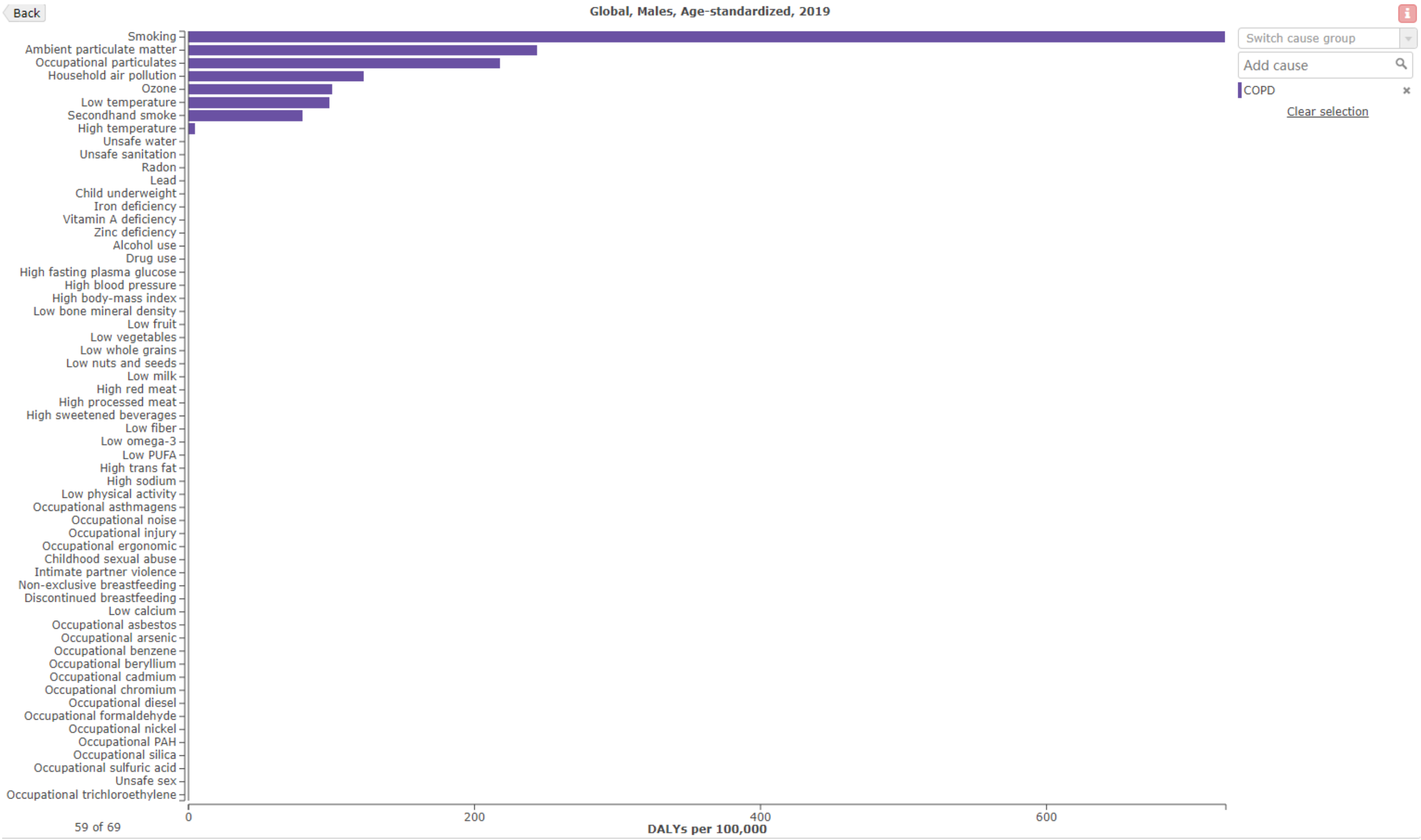

Supplementary Figure S14B: high SDI

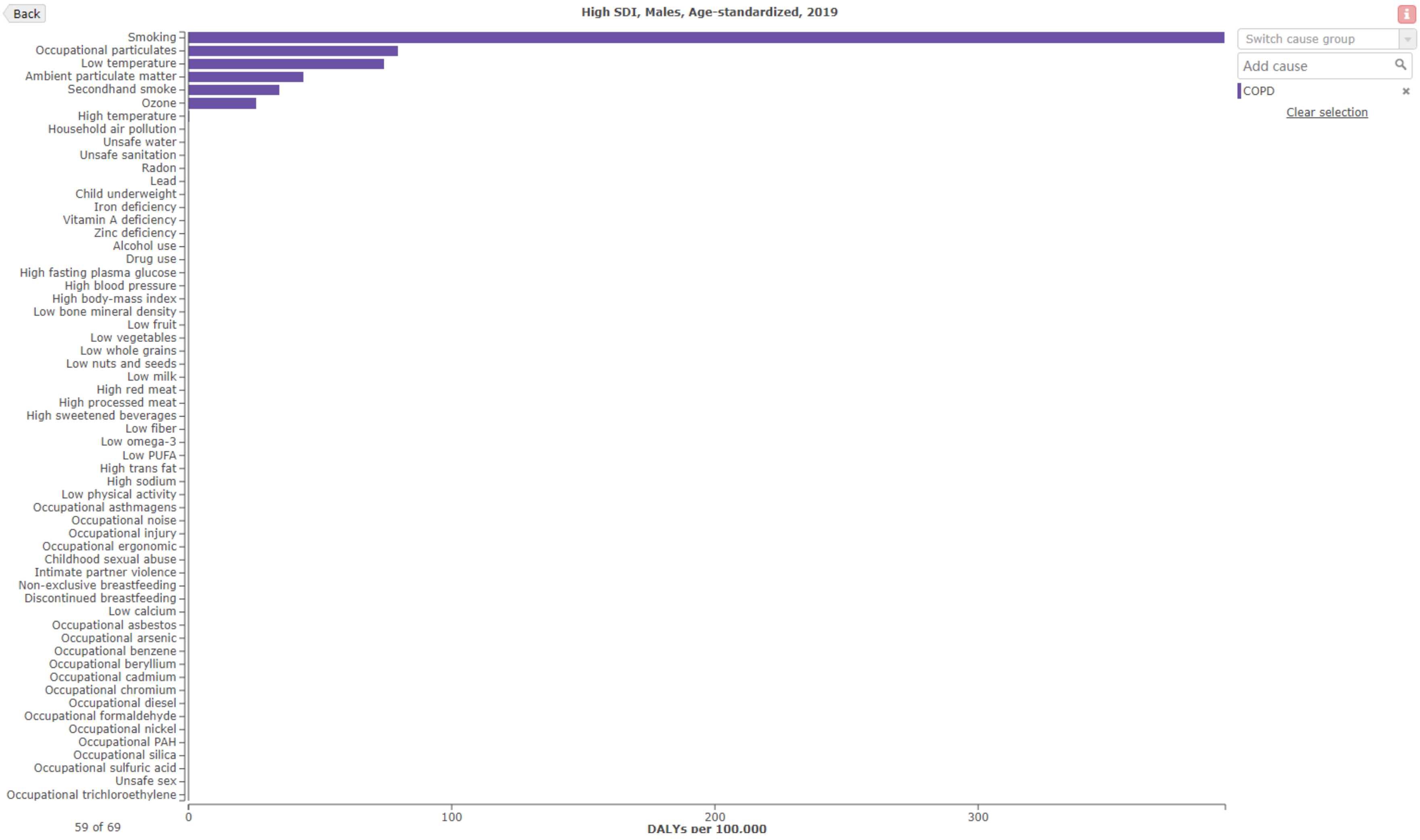

Supplementary Figure S14C: high-middle SDI

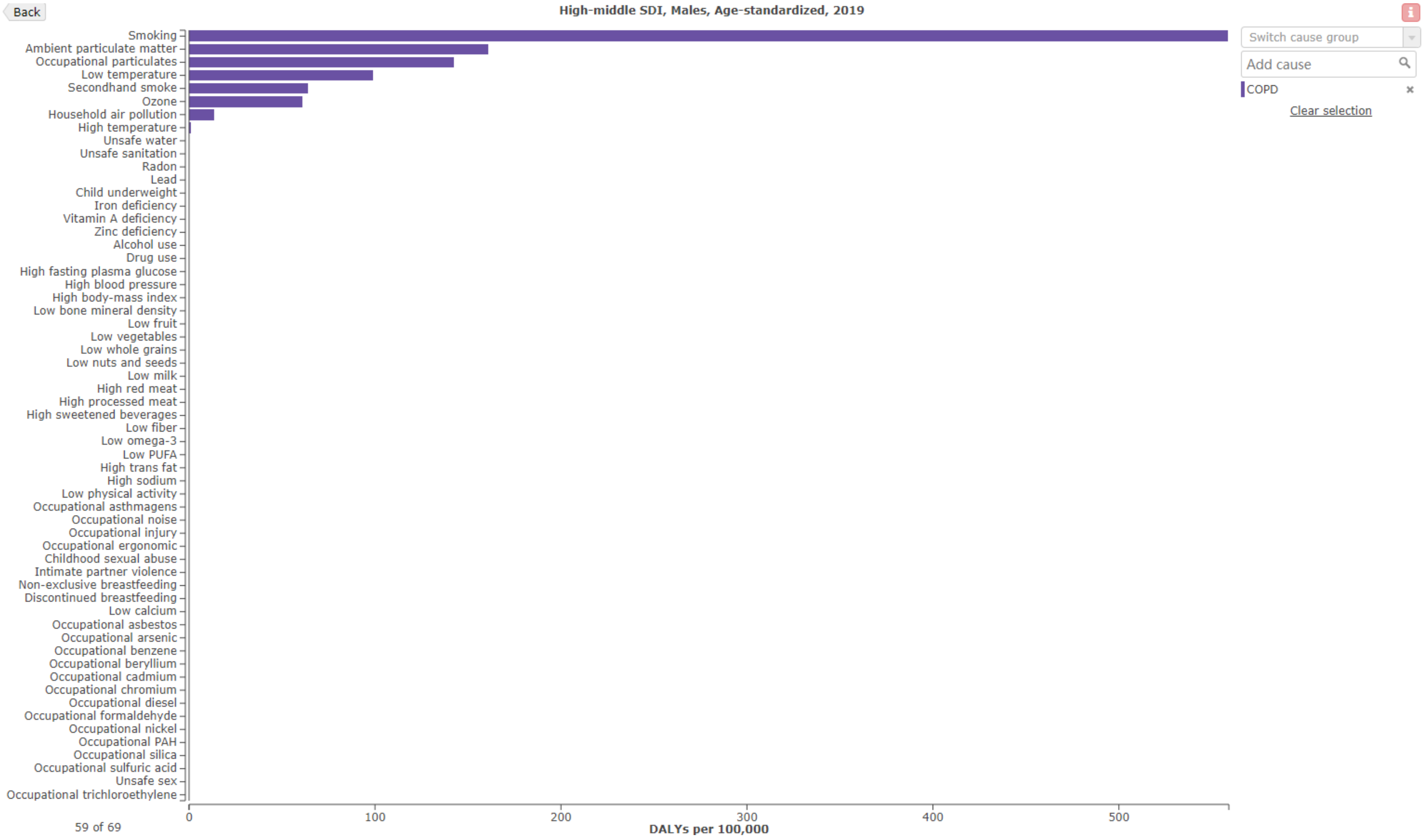

Supplementary Figure S14D: middle SDI

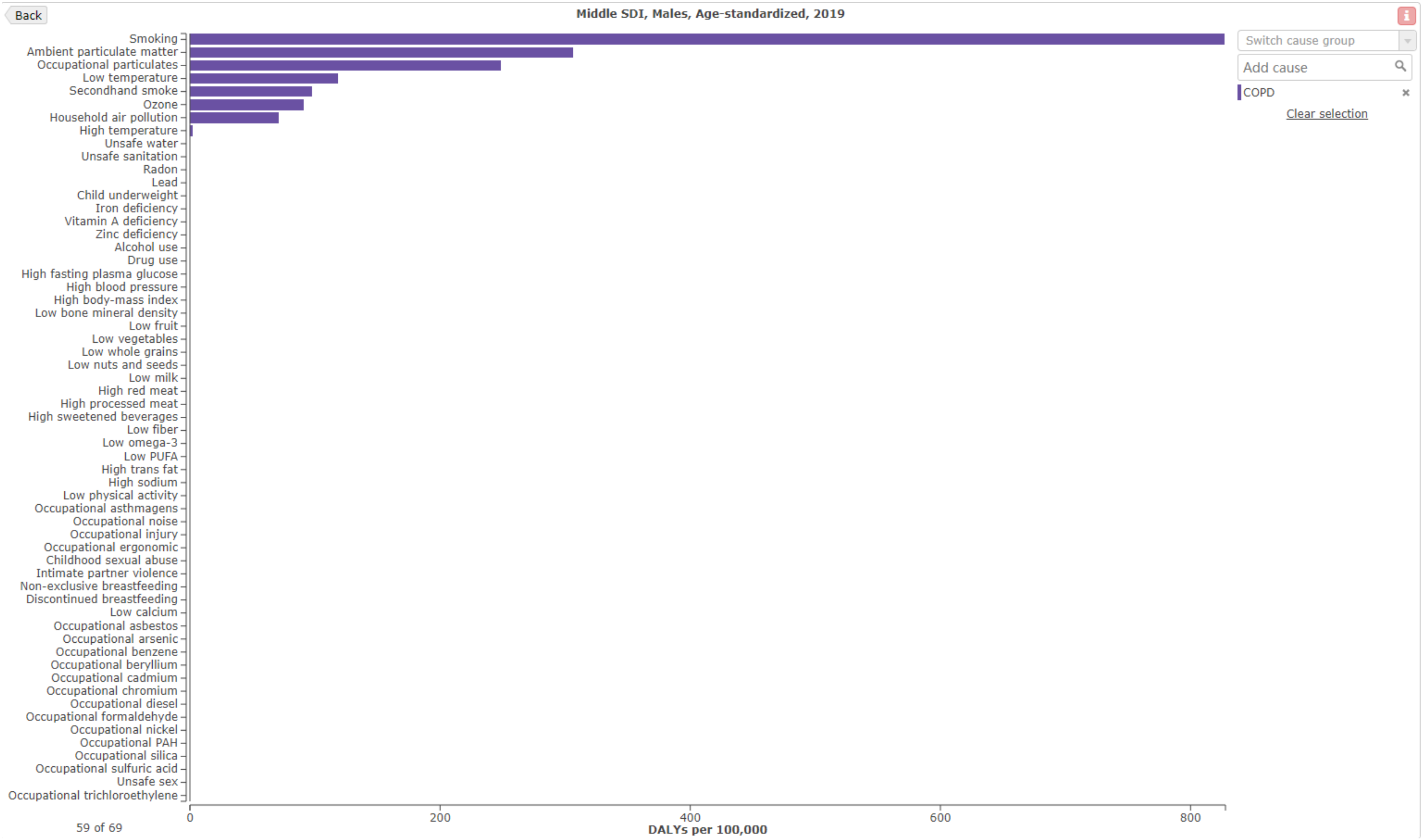

Supplementary Figure S14E: middle-low SDI

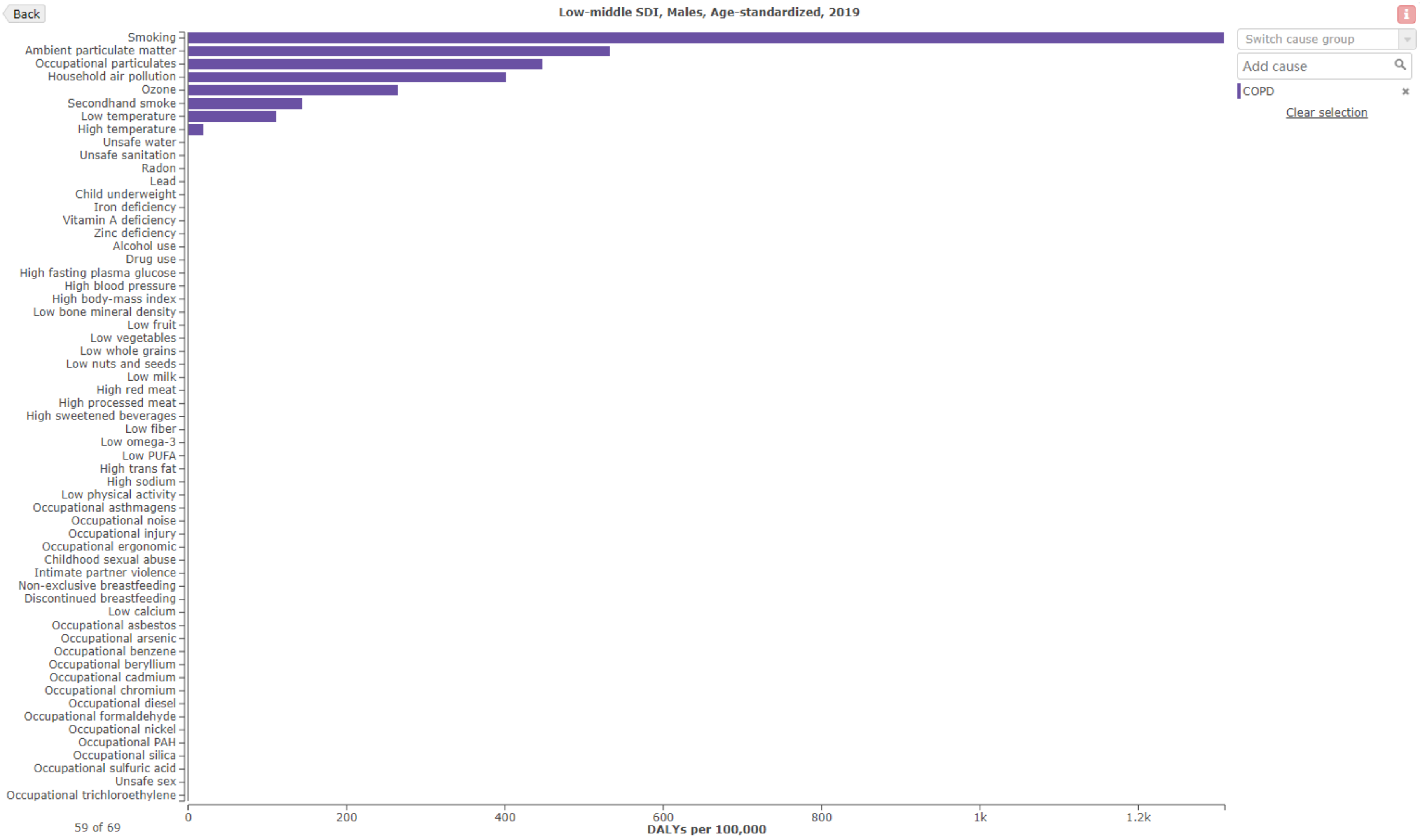

Supplementary Figure S14F: low SDI

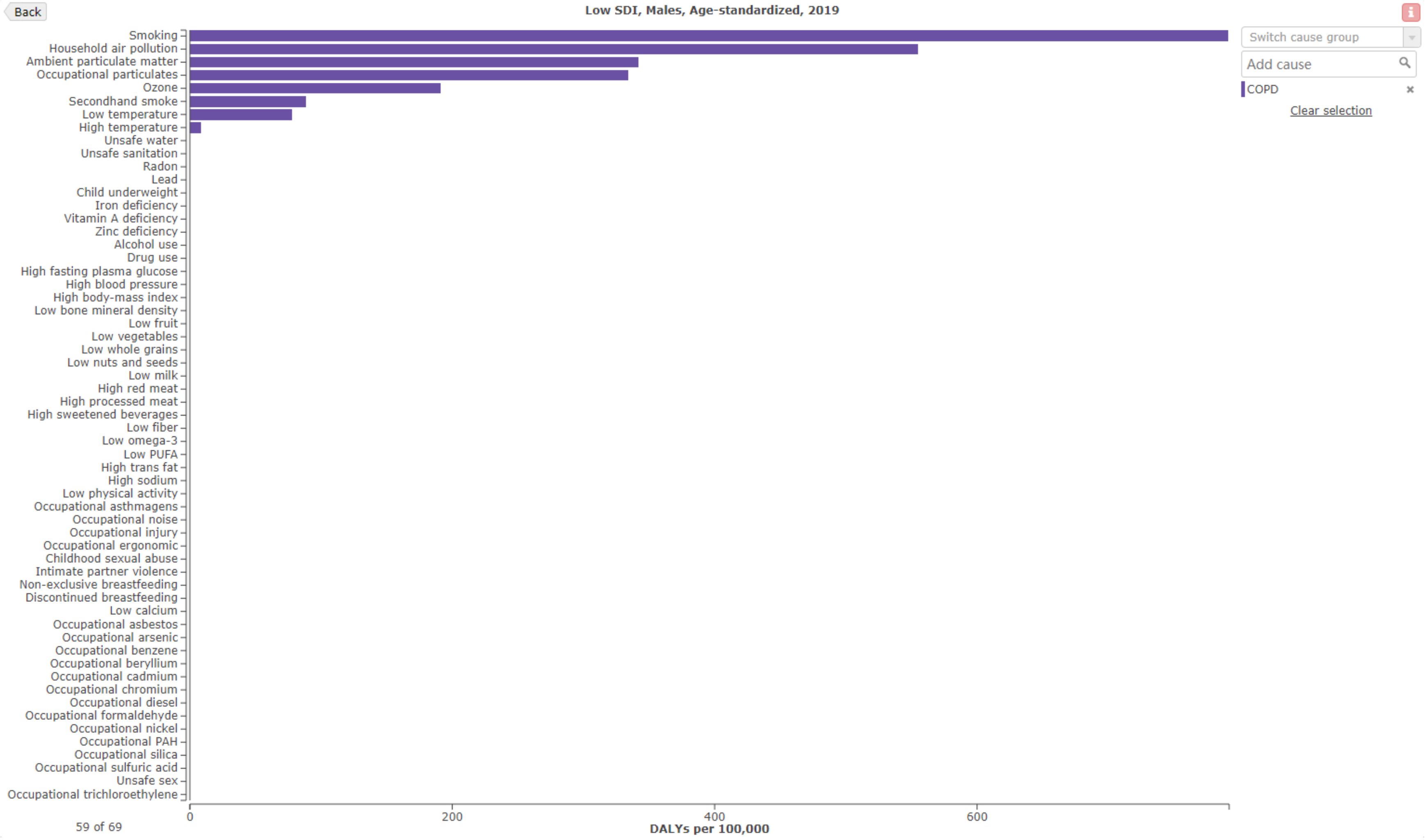

Supplementary Figure S15 The incidence, death, and DALY rates of Chronic Obstructive Pulmonary Disease in different age groups.

Note: A: Incidence in 1990. B: Incidence in 2019. C: Death rate in 1990. D: Death rate in 2019. E: DALY rate in 1990. F: DALY rate in 2019.

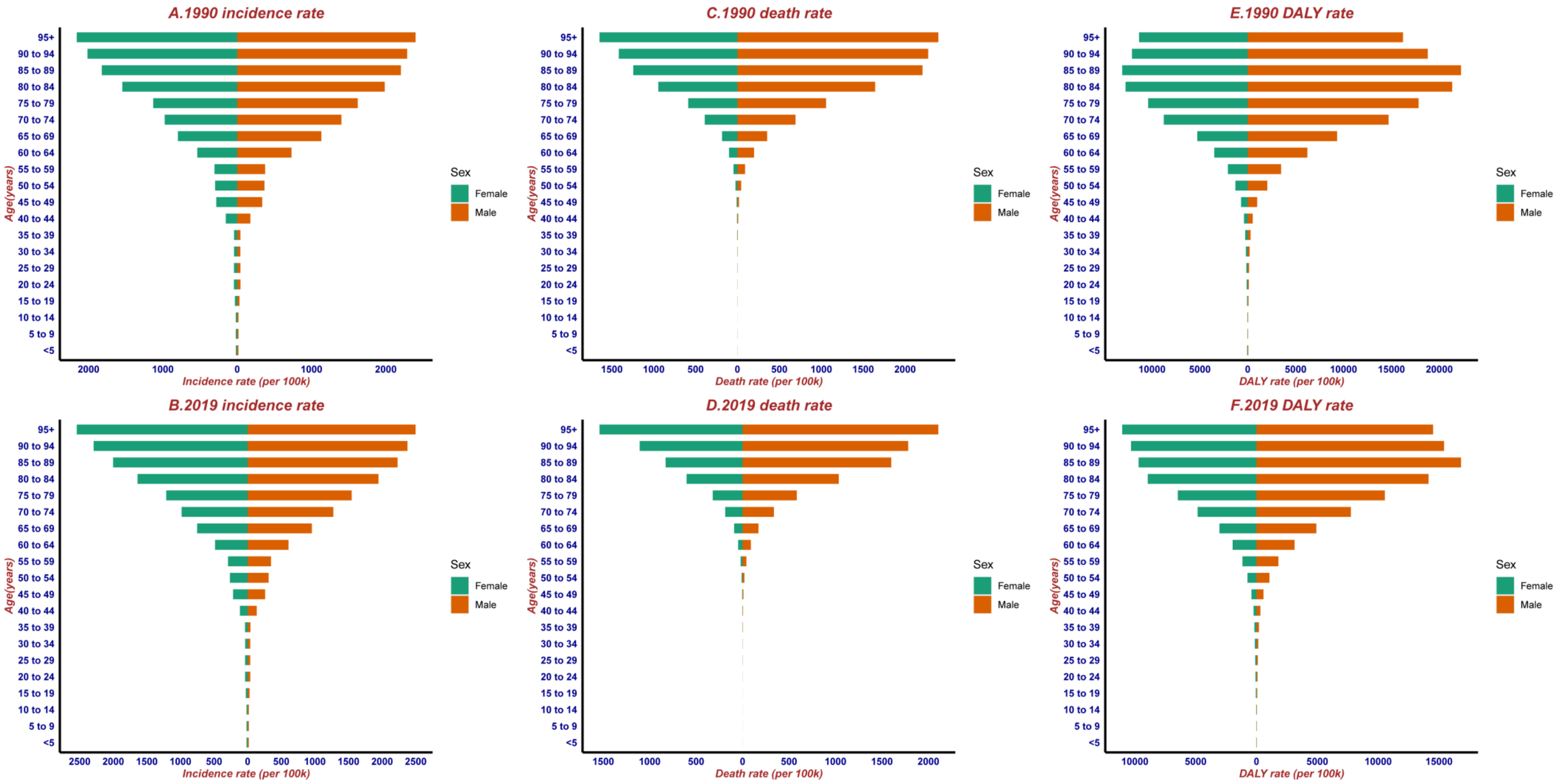

Supplementary Figure S16 The ratio of male to female age-standardized DALY rate among different age groups in global (A), high SDI (B), high-middle SDI (C), middle SDI (D), middle-low SDI (E) and low SDI (F).

Note: SDI, socio-demographic index.

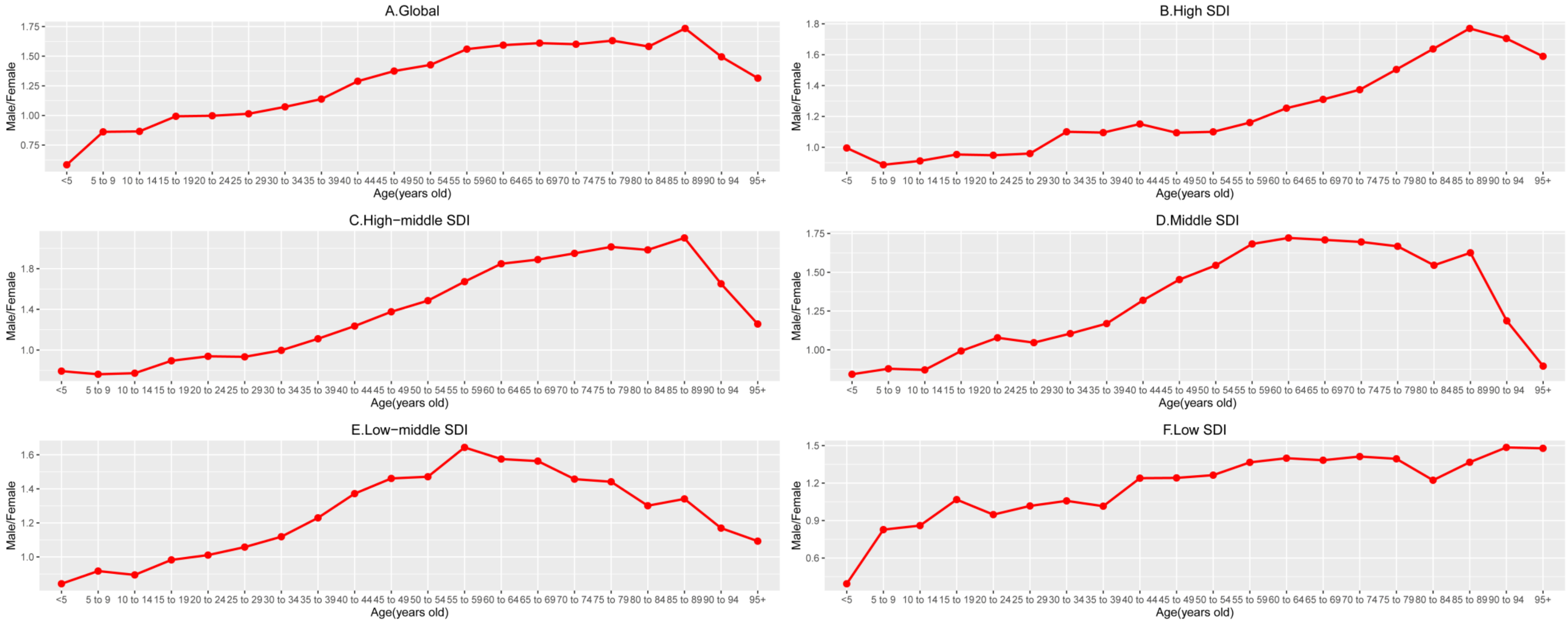

Supplement: Supplementary file 1 — Supporting Information [file RESP-28-29-s001.pdf]
